# Accounting for Solvation Correlation Effects on the Thermodynamics of Water Networks in Protein Cavities

**DOI:** 10.1021/acs.jcim.2c01610

**Published:** 2023-03-14

**Authors:** Emilia P. Barros, Benjamin Ries, Candide Champion, Salomé R. Rieder, Sereina Riniker

**Affiliations:** † Department of Chemistry and Applied Biosciences, ETH Zürich, Vladimir-Prelog-Weg 2, 8093 Zürich, Switzerland

## Abstract

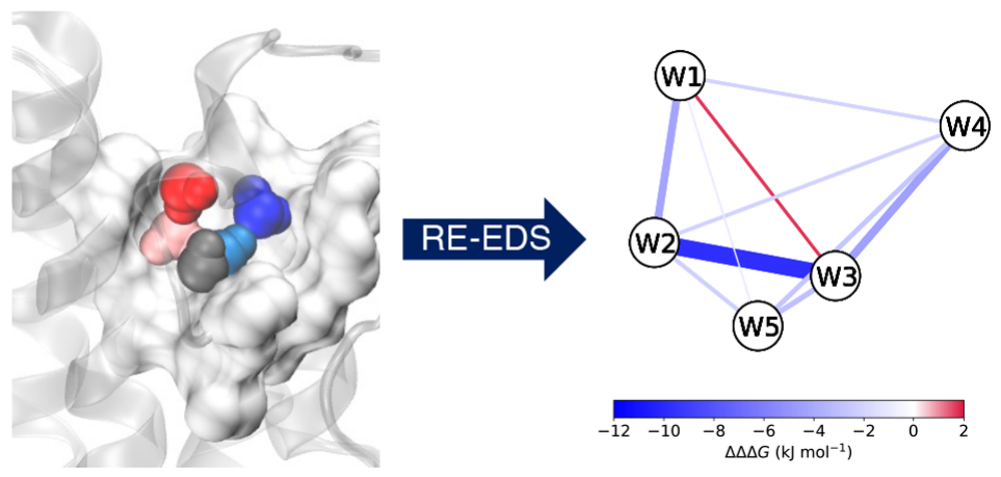

Macromolecular recognition and ligand binding are at the core of biological function and drug discovery efforts. Water molecules play a significant role in mediating the protein–ligand interaction, acting as more than just the surrounding medium by affecting the thermodynamics and thus the outcome of the binding process. As individual water contributions are impossible to measure experimentally, a range of computational methods have emerged to identify hydration sites in protein pockets and characterize their energetic contributions for drug discovery applications. Even though several methods model solvation effects explicitly, they focus on determining the stability of specific water sites independently and neglect solvation correlation effects upon replacement of clusters of water molecules, which typically happens in hit-to-lead optimization. In this work, we rigorously determine the conjoint effects of replacing all combinations of water molecules in protein binding pockets through the use of the RE-EDS multistate free-energy method, which combines Hamiltonian replica exchange (RE) and enveloping distribution sampling (EDS). Applications on the small bovine pancreatic trypsin inhibitor and four proteins of the bromodomain family illustrate the extent of solvation correlation effects on water thermodynamics, with the favorability of replacement of the water sites by pharmacophore probes highly dependent on the composition of the water network and the pocket environment. Given the ubiquity of water networks in biologically relevant protein targets, we believe our approach can be helpful for computer-aided drug discovery by providing a pocket-specific and *a priori* systematic consideration of solvation effects on ligand binding and selectivity.

## Introduction

Water molecules look deceptively simple. Despite being formed by just two elements and three atoms, these ubiquitous molecules possess an array of unique properties^1^ that make them essential for biological processes such as protein folding, enzyme function, and biomolecular recognition.^2−4^ In the context of rational drug design, there has been a growing appreciation for the role of water molecules in ligand binding,^5^ evidenced by the diversity of computational methods proposed to ensure sampling of buried water sites for accurate modeling of ligand binding interactions^6−10^ or to characterize hydration thermodynamics in binding sites.^11−13^ Targeting the displacement of water molecules in protein pockets by the addition of substituent groups to candidate molecules has been demonstrated as a successful strategy for lead optimization, resulting in increase of the binding affinity.^14−16^ However, the degree of contribution depends on a complex interplay between the water enthalpic and entropic changes upon release from the binding site, and the protein–ligand (and ligand–water) interactions that replace it.^17−20^ Depending on this balance, replacement of a binding site water may actually lead to a decrease in the ligand binding affinity.^21,22^ This often unexpected effect has led to the broad classification of water molecules into groups, with terms such as “unhappy”, “hot”, or “unstable” referring to water molecules that are energetically unstable and for which replacement is a good strategy. In contrast, “happy”, “cold”, or “stable” waters are those that contribute favorably to binding.^23,24^ In the latter case, a better strategy to improve binding affinity may be the modification of the ligand to exploit the water molecule’s beneficial contributions via, for example, the formation of water-mediated hydrogen bonds.^25,26^

Given the time and financial costs involved in lead optimization and the poor results that can arise when solvation is not properly modeled in computer-aided drug discovery,^21,27^ computational methods for hydration site profiling have increased in popularity in pharmaceutical applications.^12,28^ The *in silico* approaches can range from computationally efficient but approximate methods based on static representations of the system, such as SZMAP^29^ or 3D-RISM,^30^ to rigorous but computationally expensive alchemical free-energy methods such as double decoupling.^31^ Popular methods such as WaterMap^32^ and GIST,^33,34^ which are based on the inhomogeneous fluid solvation theory,^35,36^ lie in intermediate positions on this scale. These methods incorporate dynamic effects to some degree by the performance of short molecular dynamics (MD) simulations, although the coordinates of the protein (or at least its backbone) are typically kept fixed. Despite their methodological differences, all of the approaches mentioned above determine the stability of the water sites at a fixed composition of the environment, which is usually the apo protein pocket. This provides useful information that can be qualitatively correlated with ligand affinity, but neglects the influence of solvation correlation effects upon perturbations to the water network. In other words, an understanding of the combinatorial effect of replacement of multiple water molecules is missing. Of note, the grand canonical integration (GCI) method, based on the grand canonical Monte Carlo (GCMC) method,^37,38^ considers network cooperativity explicitly by slowly “titrating” water molecules in the binding pocket,^39^ but the stability of the water molecules is still determined at a fixed composition of the environment.

As lead optimization strategies often involve the “growth” of initial hit compounds in the pocket by addition or modification of substituents, several water molecules may turn out to be displaced or replaced by the final ligand. It is reasonable to assume that the organized hydrogen-bonding network formed by water molecules in constrained environments^40^ can lead to complex effects upon perturbation of the interactions. Water molecules predicted to be “happy” in the ligand-free pocket may thus display a completely different thermodynamic profile when nearby hydration sites are occupied by the ligand and the water becomes “trapped” inside the pocket. Indeed, computational and experimental studies point to this complex solvation pattern effect,^41−43^ which can sometimes affect ligand affinity by orders of magnitude.^22^ A systematic characterization of the network’s combined thermodynamic contributions could provide an enhanced picture *a priori* of which areas of the pocket to exploit.

**Figure 1:**
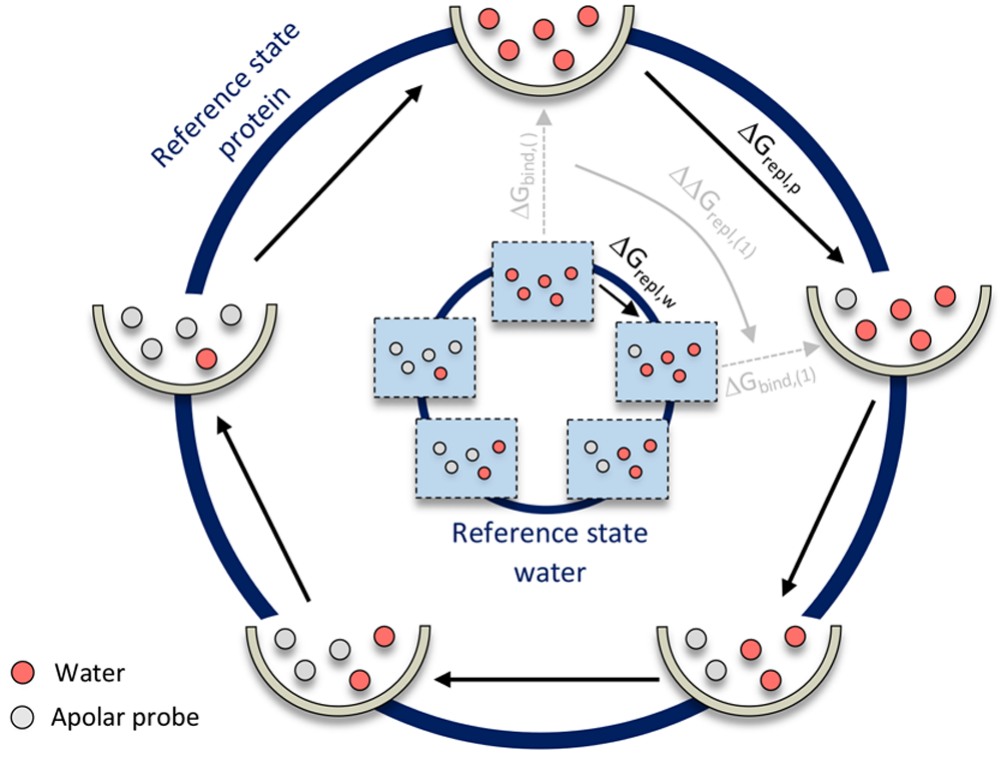
Schematic illustration of the approach to calculate free-energy differences of water replacement. End states are identified by different numbers of water (red circles) and apolar probe (gray circle) molecules. The navy blue circles represent the EDS reference state,^49−51^ such that all end states are considered simultaneously in a given environment. Sampling of the states is performed both in the protein pocket (exterior circle) and in bulk water (interior circle). For simplicity, only one of the thermodynamic cycles is represented, indicating the free energies of replacement of water at position 1 in the protein pocket and bulk water (Δ*G*_repl,p_ and Δ*G*_repl,w_, respectively). The dashed arrows indicate the absolute binding free energy of each water/probe network composition, identified in parentheses according to the hydration site ID occupied by a probe. From the thermodynamic cycle, the relative free energy of replacement of the hydration sites can be calculated (ΔΔ*G*_repl,(1)_). If all combinations of replacements to the water network are investigated, more states than represented are simulated.

In this study, we investigate the conjoint effects of replacing multiple groups of water molecules in binding pockets to better characterize their influence on ligand binding. We take advantage of the computational efficiency of the replica-exchange enveloping distribution sampling (RE-EDS) multistate method^44,45^ to explicitly calculate the free energies of water molecule replacement via rigorous free-energy calculations (Figure 1). This method has been previously applied to the calculation of ligand binding and small-molecule solvation free energies.^44−48^ Here, we extend RE-EDS to enable the quantification of water thermodynamics based on the free energy of replacing water sites by apolar “pharmacophore” probes.^41^ Validation results on a three-water network in the small bovine pancreatic trypsin inhibitor (BPTI) protein evidence the degree of solvation correlation effects on water stability, with reductions of up to 16.5 ± 2.2 kJ mol^–1^ on the calculated free-energy differences when correlation effects are taken explicitly into account. Variations of up to 11.2 ± 0.8 kJ mol^–1^ on the favorability of replacement of the same water molecules can be observed upon changes to the network environment. Application of the method to the conserved water network of four bromodomain proteins shows qualitative agreement with known inhibitors of these often difficult to target proteins. In support of previous observations, our results highlight the existence of different water stability profiles and network correlations within the bromodomain family, suggesting opportunities for selective inhibitor design.

## Theory

A general overview of the RE-EDS method is given here, with emphasis on the interpretation of the EDS states and the parameters necessary for the reference state construction. Interested readers are referred to the Supporting Information for more detailed discussions of the mathematical basis of the method and the procedure adopted for calculation of free-energy differences of water replacement.

### Free-Energy Methods: EDS and RE-EDS

**Figure 2:**
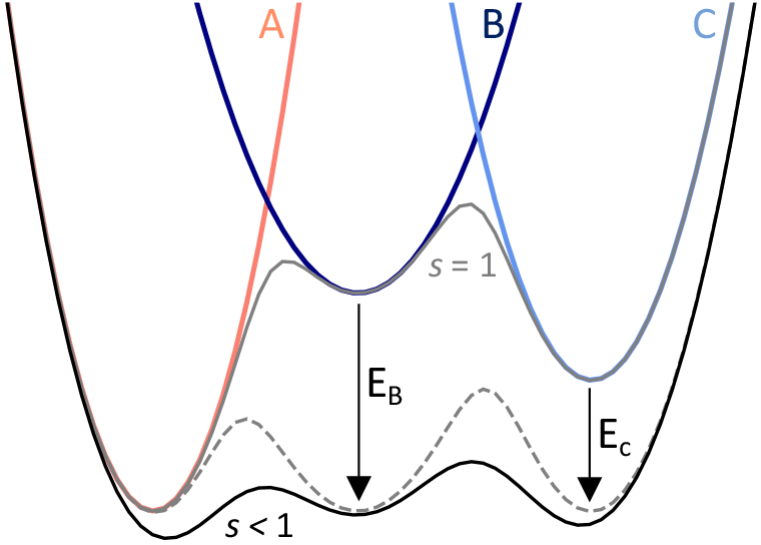
Representation of the potential-energy landscape of three end states *A*, *B*, and *C* (in pink, navy and light blue, respectively), and the EDS *s* and  parameters that are tuned to ensure equal weighting of the end states in the reference potential (gray and black curves, respectively).

The EDS free-energy method allows for the calculation of free-energy differences between multiple end states from a single simulation by the construction of a reference state Hamiltonian that envelops the Hamiltonians of the end states (Figure 2).^49−52^ Two sets of parameters, the smoothing parameter *s* and the energy offsets , are tuned to enable optimal sampling of the end states.^46,49,50,52^ The smoothing parameter decreases the energy barriers between the potential-energy minima corresponding to each end state, such that transitions can occur within the reference state, while the energy offsets align the minima of the potential-energy surface of the end states such that all contribute equally to the reference potential-energy landscape.

In the recently developed replica-exchange EDS (RE-EDS) method,^44,45^ the parameter search has been simplified through the introduction of Hamiltonian replica exchange in the *s* space, thus reducing the search space to the selection of an appropriate range and distribution of the *s* replicas, together with the estimation of the energy offsets. An automated pipeline has been developed for the estimation and optimization of these parameters for RE-EDS calculations,^46^ and details of the adaptations introduced for the estimation of water replacement free energies are provided in the Supporting Information (Theory and Figures S1–S5). Of particular importance, nearly equal sampling of all states by the reference state is ensured by tuning the energy offsets in an iterative  rebalancing step.

The final free-energy differences between each pair of states is then calculated from the physical *s* = 1 replica by the application of Zwanzig’s equation^53^ twice.^49,50^

### End State Definition

**Figure 3:**
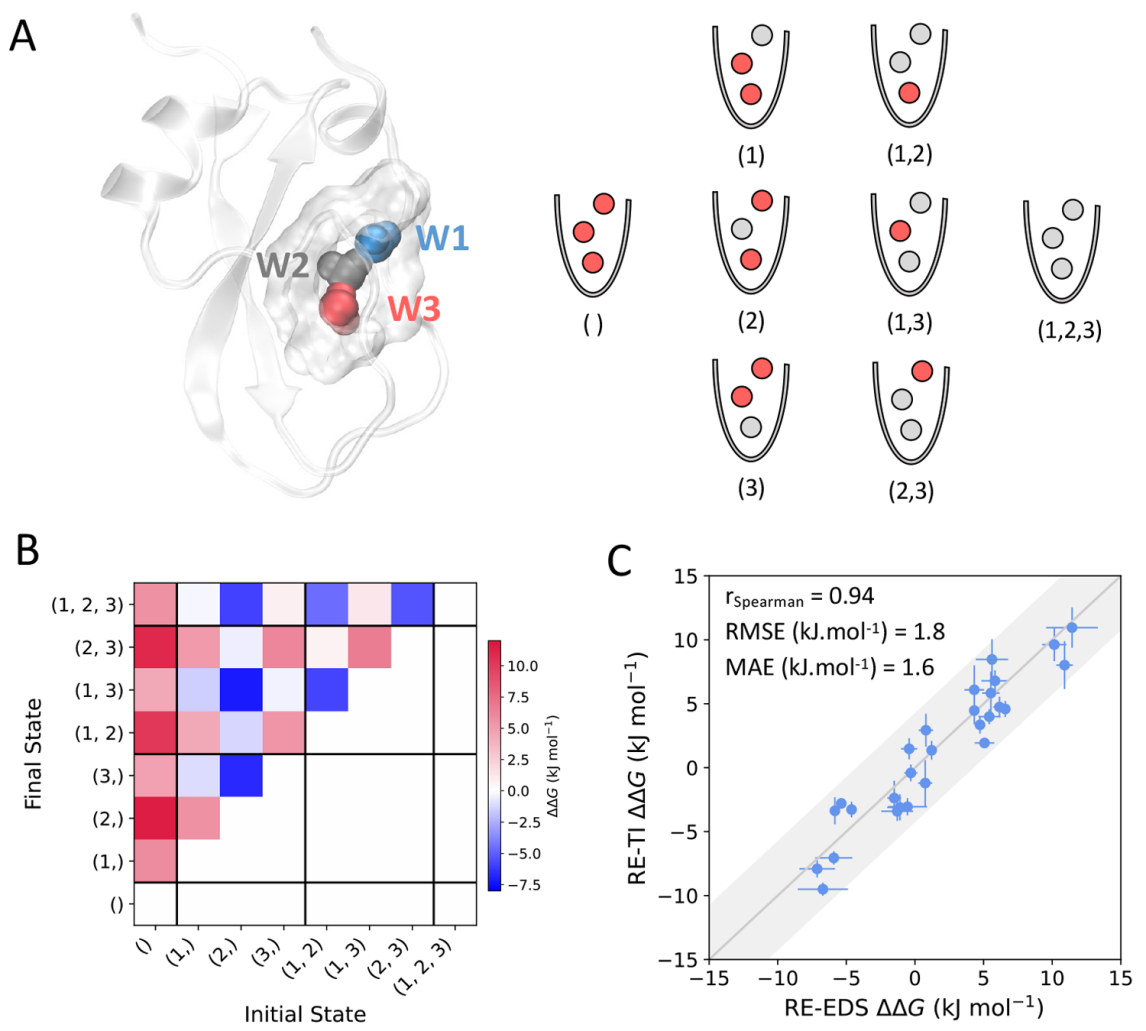
RE-EDS results for water replacement in the bovine pancreatic trypsin inhibitor (BPTI) cavity containing three water molecules. (A) BPTI structure with the three waters considered (left) and representation of the eight states considered upon combinatorial replacements of the water molecules (red circles) by apolar probes (gray circles, right). States are labeled according to the hydration site ID occupied by a probe. (B) ΔΔ*G*_replacement_ for each pair of initial and final states, averaged over the triplicate protein and bulk simulation runs. (C) Comparison of the RE-EDS and replica-exchange thermodynamic integration (RE-TI) results. Shaded area indicates deviation of ±1 kcal mol^–1^. Error bars denote standard deviations, calculated by error propagation of the water and protein triplicate’s standard deviations.

The end states in small-molecule relative binding and solvation free-energy calculations with (RE-)EDS are very clearly defined, as each state corresponds to one of the molecules of interest. In analogy to previous work using EDS for water thermodynamics calculation,^41^ each end state here is defined as a combination of water and probe molecules, in which the probe molecules replace a specific number of the waters in the network under investigation. In this work, since we want to investigate the extent of solvation correlation effects on the energetics of water molecules and test the robustness of the parameter estimation procedure for a large number of states, we considered all possible combinations of replacements to the water network (Figure 3a). The probe used is a CH_3_ group with a neutral charge (using the GROMOS united atom representation,^54^ this corresponds to a single particle of zero charge), as a mimic of an apolar aliphatic substituent introduced to a growing scaffold during lead optimization.^41^

The perturbations to the water network are performed in two separate simulations, one in the protein pocket (external circle, Figure 1) and one in bulk water (internal circle, Figure 1), using weak position restraints in both cases to localize the alchemical water molecules. The relative free-energy difference between each pair of states in these simulations thus yields the free energy of replacement of the water molecule in the protein pocket compared to the same perturbation in bulk, ΔΔ*G*_replacement_, or in other words, the binding free energy of the apolar probe to that hydration site (see for example the thermodynamic cycle represented in Figure 1). The position restraints are kept constant in both bulk and protein pocket simulations, with the assumption that their contribution is canceled via the use of the thermodynamic cycle.

## Results and Discussion

### Validation on a Small Cavity

The small bovine pancreatic trypsin inhibitor (BPTI) protein was chosen as the test system to validate the RE-EDS workflow for the calculation of water replacement free energies. This well-studied protein contains a narrow, buried three-water cavity that was the subject of another computational method for water thermodynamic profiling, suggesting cooperativity in the hydration of the cavity.^39^ Considering the substitution of all possible combinations of the water molecules by apolar probes, this three-water cavity leads to the definition of eight RE-EDS states: one all-water state, three states in which each of the waters is replaced by a probe, three states with two-water replacements, and a final all-probe state (Figure 3a).

The initial energy offsets, defined based on the number of apolar probes in the state (see Theory, Supporting Information), were optimized quickly in the rebalancing step for the simulations in bulk water (Figure S6), leading to nearly ideal sampling of all states in as few as two iterations (Figure S4a). The triplicate simulations in the protein pocket required more iterations, which is to be expected in this more slowly adapting environment. Equal sampling of all states is harder to reach in this case, as evidenced by the larger deviations from ideal sampling (Figure S4b). In both simulations, the changes to the energy offsets during the rebalancing iterations improved not only state sampling but also the number of roundtrips between replicas, reducing the average roundtrip times (Figure S7), since these two parameters are correlated. The results indicate that, if the replica distribution in *s* parameter space is sufficiently good to ensure at least some exchanges between replicas, a more efficient use of the computational resources lies in performing iterations of energy offset rebalancing, as this step can compensate issues due to a possible suboptimal replica distribution.

The deviation from equal (ideal) state sampling was used as stop criterion for rebalancing and to start production simulations (Figure S4). Convergence analysis indicate that 1.8 and 4.2 ns are enough sampling for the solution and protein simulations, respectively (Figure S8). The ΔΔ*G*_replacement_ between all combinations of end states, resulting in a total of 28 values (Table S4), are displayed in Figure 3b and show very good agreement with values calculated with replica-exchange thermodynamic integration (RE-TI, Figure 3c). However, because these are calculated from a single set of RE-EDS simulations, instead of individual pairwise perturbations as with RE-TI, the RE-EDS method is much more computationally efficient (Table 1). The RE-TI protocol adopted was computationally intense in order to ensure converged results and was performed for all pairwise calculations, such that it constitutes an upper limit of the estimated computational cost. For comparison, an estimate for a lower limit is provided with hyperparameters similar to those seen in the literature (11 λ windows, 5 ns each for the protein simulations, and 0.5 ns for solution simulations). The choice of hyperparameters is of course highly system dependent, and therefore, any lower and upper limits can only be estimates.^55−57^ For the calculation of this lower limit, we also considered the minimum number of pairwise calculations (*N* – 1) and disregarded any equilibration steps.

**Table 1:** Cumulative Simulation Time Required for Converged Results of RE-EDS and RE-TI Calculations Performed in This Study (RE-TI upper limit) and an Estimate of a Lower Limit for the RE-TI Calculations^a^

			RE-TI	
Simulation	Sampling time (ns)	RE-EDS	lower limit	upper limit
Solution	*t*_preparation_	33.6	–	294
	*t*_production_	37.8	38.5	294
Protein	*t*_preparation_	84	–	294
	*t*_production_	88.2	385	5880
	Total	243.6	423.5	6,762

a This lower-limit estimate was calculated for *N* – 1 pairwise calculations with 11 λ windows, 5 ns each for the protein simulations, and 0.5 ns for solution simulations, disregarding equlibration steps.

### Effects of Pocket Occupancy on Water Stability

**Figure 4:**
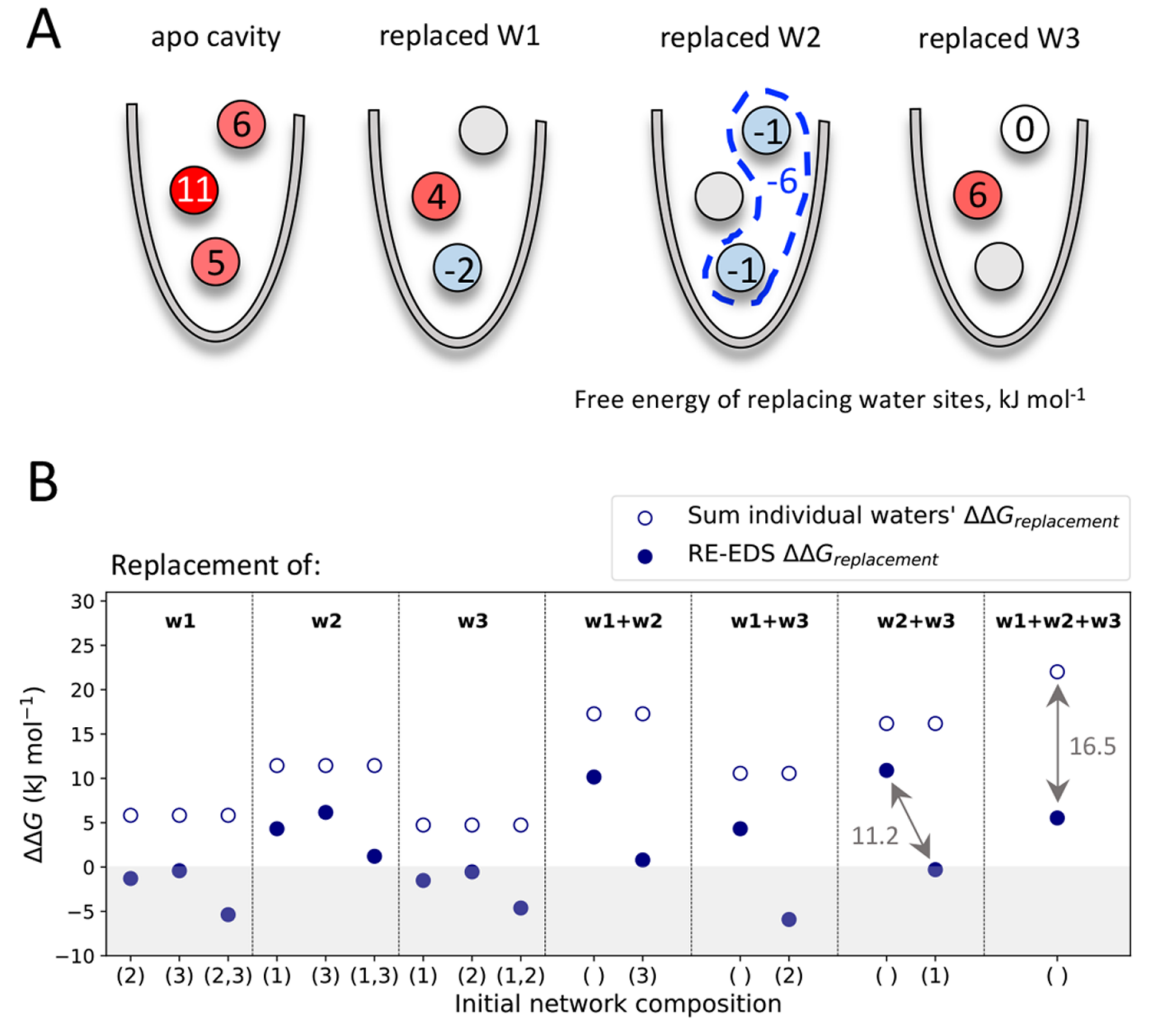
Changes in water “happiness” upon alterations of the BPTI water network. (A) Schematics of the free-energy cost for replacement of the indicated water molecules (ΔΔ*G*_replacement_ values in kJ mol^–1^), at different initial pocket environments. (B) Comparison of ΔΔ*G*_replacement_ for the indicated water molecules (full circles), against the sum of the individual waters’ ΔΔ*G*_replacement_ calculated from an apo pocket, neglecting solvation correlation effects (blank circles). Arrows indicate the largest changes in replacement free energies upon alterations to the network and explicit consideration of correlation effects. Distinct water/probe network compositions are indicated by the hydration site ID’s occupied by a probe.

The use of weak position restraints on the oxygen atoms of the alchemical water molecules allows for their localization into occupancy volumes (Figure S9) and the interpretation of the results in terms of the replacement of the hydration sites by apolar probes. Individual replacements in the apo pocket of BPTI indicate that replacing water molecules 1, 2, or 3, when the nearby hydration positions are occupied by water, is highly unfavorable: ΔΔ*G*_replacement_ values are 5.8 ± 1.0, 11.4 ± 1.9, and 4.7 ± 0.2 kJ mol^–1^, respectively (bottom results in the first column of the free-energy matrix in Figure 3b and represented schematically in Figure 4a). The higher cost of replacing W2 can be attributed to its central position in the H-bonding network, leading to a highly unfavorable disruption of the network. However, these perturbations become less unfavorable upon changes to the pocket environment: the free energy of replacing W1, for example, not only decreases but becomes negative if W2 or W3 have been replaced first (Figure 4a) and is even more favorable if both positions 2 and 3 are initially occupied with apolar probes as it leads to a fully apolar final state (Figure 4b). The same trends are observed for replacement of W2 and W3, with W2 retaining its overall higher free-energy costs of replacement.

The results in Figure 4 evidence not only the decrease in stability of the remaining water molecules as the nearby environment changes, making replacements more favorable, but also the existence of cooperativity within the network. For example, concomitant replacement of water molecules 1 and 3 when W2 is already replaced (leading to a fully apolar cavity) is much more favorable than the sum of the free energies of perturbing each water molecule individually (Figure 4a, dashed dark blue line). In fact, replacement of any combination of water molecules, including concomitant pair or triple replacements, always leads to a lower ΔΔ*G*_replacement_ than the sum of the free energies obtained from individually targeting the water molecules in the apo cavity (see difference in value of the full and blank circles, Figure 4b), emphasizing the nonadditivity of these terms and the existence of solvation correlation effects.

Water stability in the same three-water cavity of BPTI has been computationally studied with the grand canonical integration (GCI) method,^39^ in which water molecules are “titrated” into the empty cavity, and the location and energetics of the waters can be tracked as an incremental amount of water molecules are inserted in the pocket. Although this is not the same process as simulated with RE-EDS (we replace the water molecules with the apolar probe instead of removing it completely), the GCI results also indicate cooperativity effects between the water molecules, in line with our findings.

### Toward Larger Network Perturbations

We next sought to test the robustness of the method by applying it to the more challenging and biologically relevant bromodomain protein targets. Bromodomains are epigenetic readers that recognize acetylated lysine residues on histones and other proteins.^58^ Due to their role in the regulation of gene transcription, they have attracted considerable interest as promising drug targets for treatment of a variety of diseases, such as cancer, inflammation, and heart failure, with several compounds in clinical trials.^59^ However, the high structural similarity and binding pocket sequence conservation exhibited by the 61 members of this protein family present a significant challenge to the design of selective inhibitors. Inhibitors of the most widely studied bromodomain, BRD4(1), for example, often also bind to the first bromodomain of the other Family II proteins BRD2(1), BRD3(1), and BRDT.^60^ Even intraprotein selectivity has been challenging to achieve, as indicated by the tremendous effort required to design ligands that differentiate between the first and second bromodomains of BRD4 (BRD4(1) and BRD4(2)).^61−65^

**Figure 5:**
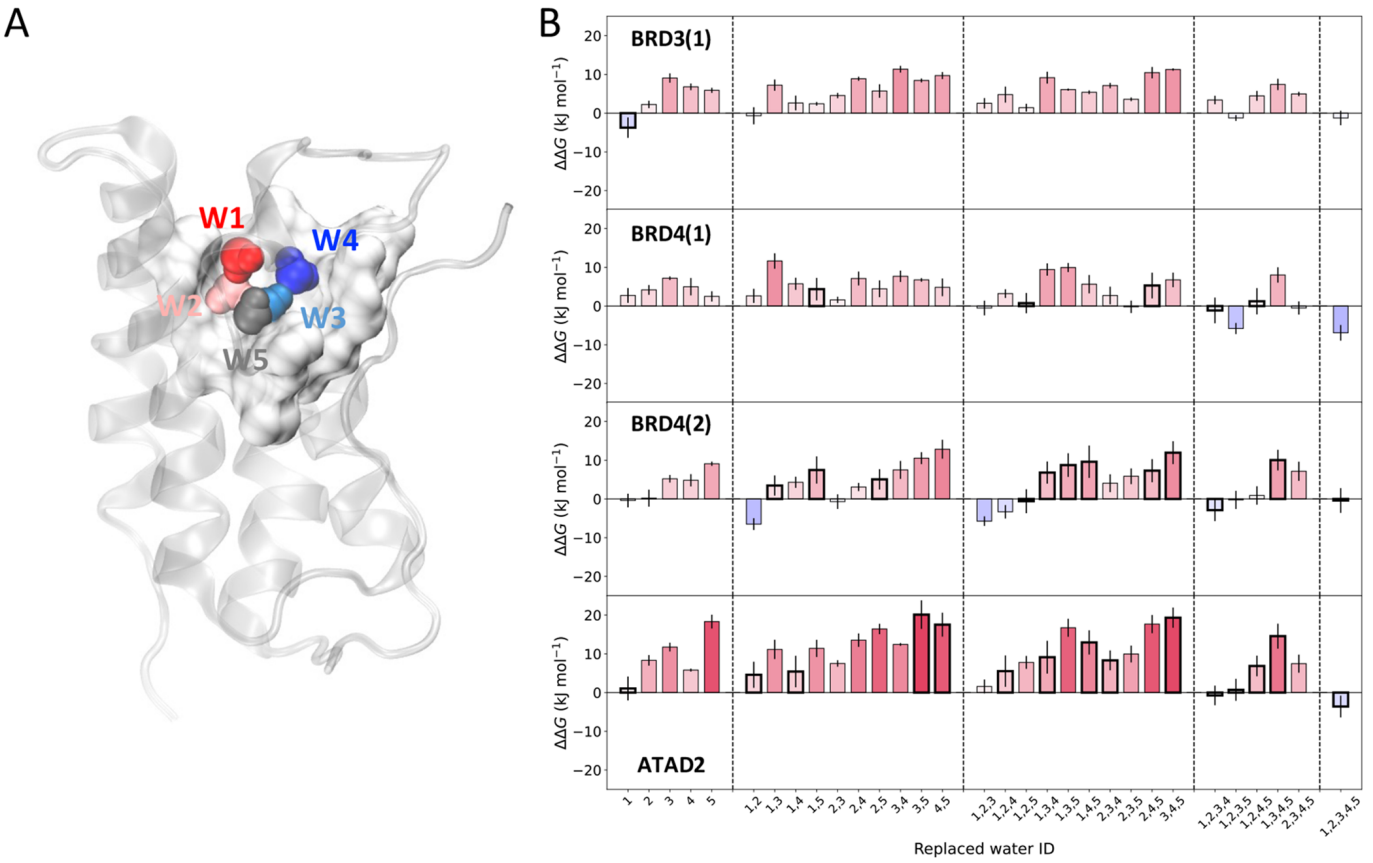
(A) Representation of the bromodomain five-water network considered in the RE-EDS calculations. (B) Free energies of replacing all combinations of water molecules from the apo cavity. Error bars indicate the associated standard deviations. Bars with thick edges indicate data points for which the standard deviation is above 2.5 kJ mol^–1^. Dashed vertical lines separate single, pairwise, and higher order concomitant replacements.

These small protein domains exhibit four antiparallel α-helices connected by loop regions, forming a deep hydrophobic pocket. Crystallographic structures highlight the existence of a conserved network of water molecules located at the bottom of the pocket (Figure 5a). Perturbation of this network by the insertion of ligand substituents has proven to be extremely difficult. However, recent computational works suggest that the stability of the network differs among members of the family,^66,67^ paving a promising strategy for the design of selective bromodomain inhibitors. Indeed, targeting of conserved water molecules is a determinant factor for selectivity in kinases^68,69^ and is supported by the selectivity of the few known bromodomain inhibitors that displace or exploit the water network.^65,70,71^

Given the experimental and computational evidence indicating different stabilities for the conserved water network in bromodomains, we decided to investigate the water replacement profiles of four of these proteins: Family II’s BRD3(1), BRD4(1), and BRD4(2), which have high levels of similarity in sequence and structure, and the more distinct Family IV’s ATAD2.^72^ Again, we consider all possible perturbations to the network by replacing every combination of the five water molecules with apolar probes, which leads to a total of 32 states to be sampled by RE-EDS.

Initial simulations with the same conditions as for BPTI indicated a large degree of flexibility of the ZA-loop located above the cavity entrance, in accordance with previous MD simulations.^73,74^ To reduce disturbances to the water network by transient insertion of loop residue side chains into the pocket, while still retaining a flexible description of the protein, we decided to increase the force constant of the position restraints that localize the oxygen atoms of the selected water molecules. The waters are still weakly positioned restrained, able to not only rotationally reorient themselves but also explore a small local volume within the cavity, but are less sensitive to the flexibility of the loop (Figure S10). We believe that this can be a good strategy for handling flexible proteins without the need to completely fix the backbone motion.

Despite the significantly higher number of end states, the RE-EDS pipeline was able to estimate the parameters necessary to ensure nearly ideal sampling of the states (Figure S11, where ideal sampling corresponds to the situation in which all end states are sampled for an equal proportion of the simulation time; see the definition in the Supporting Information Theory section). Again, in this case, convergence is much quicker for the bulk simulations. The use of triplicate energy offset rebalancing steps with different intensities of the adjusting parameter (see Theory, Supporting Information) led to distinct state sampling distributions in the protein simulations, which can compensate poor sampling of specific states in each of the replicates. This leads to the calculation of 496 unique relative free-energy results from the production simulations, as well as a measure of the degree of confidence on the results based on the spread of the values among the three replicates (Figures S12–S15). These 496 values correspond to the pairwise ΔΔ*G*_j,i_ between each of the 32 states (a matrix of 32 × 32 values), disregarding the 32 cases when *j* = *i* (in which case ΔΔ*G*_j,i_ = 0), and half of the remaining values since ΔΔ*G*_j,i_ = −ΔΔ*G*_i,j_

### Conserved Waters of Bromodomains Have Distinct Energetic Profiles

By selecting starting conformations for RE-EDS that showed good overlap with the resolved water network coordinates, and the use of the position restraints, we can ensure a high degree of equivalency between the alchemical waters in each of the studied bromodomains, such that results for waters 1 to 5 can be directly compared across the systems (Figure S10). The volume occupancies of the water molecules may not show a complete overlap between the replicates and the targets due to variations in the protein ensemble visited during the simulations, but the relative positions of the waters in the network remain constant among the systems such that the ΔΔ*G*_replacement_ results can be directly compared.

**Figure 6:**
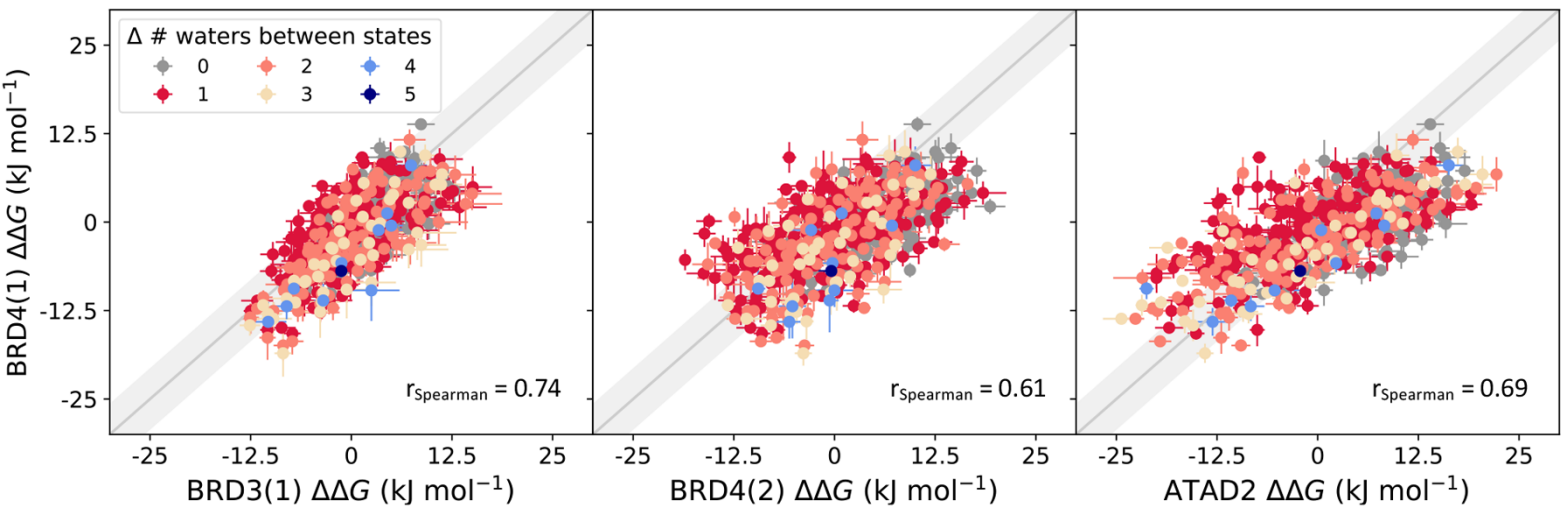
Comparison of bromodomain water energetic profiles. ΔΔ*G*_replacement_ data points are colored according to the difference in the number of water molecules present in the respective initial and final states. Spearman correlation coefficients are shown. All correlation coefficients have an associated *p*-value of 0.0002, calculated via permutation test (see the Supporting Information, Analysis section), indicating that the correlations are statistically significant.

The results shown in Figure 6 indicate that the bromodomains have distinct water thermodynamic profiles, in accordance with previous findings.^66,67^ Interestingly, the correlation of the replacement free energies of the selected bromodomains with that of BRD4(1) follows their sequence and structural similarities. Among the set, BRD3(1) is the most similar to BRD4(1), exhibiting 65.4% overall sequence identity, 90% identity of the binding pocket residues (residues located within 5 Å of the water network), and 1.46 Å root-mean-squared deviation (RMSD) based on the crystal structures. This similarity is replicated in the high correlation of the water ΔΔ*G*_replacement_ profiles, in agreement with the general difficulty of achieving intrafamily selectivity.

While still sharing a high degree of structural similarity with BRD4(1), the second bromodomain of BRD4 is more distinct to it than BRD3(1) is (34.4% whole protein sequence identity, 81% binding pocket identity, and RMSD of 1.63 Å to BRD4(1)). In accordance, we obtain a lower correlation with the water replacement profiles of BRD4(1), with a larger spread in the results (middle panel in Figure 6). Finally, ATAD2, being the most distinct of the proteins (18.5% whole protein sequence identity, 58% binding pocket identity, and RMSD of 2.1 Å to BRD4(1)), also exhibits the most distinct distribution of ΔΔ*G*_replacement_ values (right panel in Figure 6). The correlation, as measured by Spearman’s ρ, is actually higher than that of BRD4(2), but the energy values are offset from the *x* = *y* diagonal. Still, the spread of the ATAD2 data was smaller than initially expected considering its relative distance to the Family II’s proteins in the bromodomain phylogenetic tree.^72^ We hypothesize that the relatively small distinction between the BRD4(2) and ATAD2 distributions are due to compensating effects of pocket sequence and shape/dynamics on water thermodynamics. While ATAD2 has the most distinct pocket sequence, BRD4(2) shows a larger deviation in pocket volume compared to BRD4(1) (+37% versus −5%, based on the crystal structures), which is also visible in the wider BRD4(2) distribution of pocket volumes during the simulations (Figure S16). This emphasizes how the local pocket shape is also an important factor affecting water thermodynamics.

A very large number of free-energy values are obtained in the RE-EDS calculations (Figures S12–S15), including perturbations that may not be necessarily interesting from a lead optimization perspective, such as the “jumping” of a probe between hydration positions. Therefore, we decided to investigate a selection of the results in more detail, which describe the favorability of the perturbations to all combinations of water molecules starting from the apo (water-filled) cavity. Results are shown in Figure 5b, where again different profiles are observed for the studied bromodomains. Numerical values can be found in Table S5 in the Supporting Information.

For single water replacements, all of the bromodomains indicate a preference for replacement of W1. BRD3(1) and BRD4(2) in fact show favorable or negligible costs for the replacement of this water molecule, respectively (although the error in the BRD3(1) prediction is above our selected confidence limit of 2.5 kJ mol^–1^). In contrast, W5 is particularly less stable in BRD4(1), indicated by the lower cost for replacement at that site compared with the other proteins. When joint perturbations to the water network are considered, larger distinctions among the proteins emerge. In general, the members of Family II exhibit lower costs for partial replacement of the water network. The more distinct ATAD2, on the other hand, exhibits a much higher cost for partial network replacement. The replacements are only favorable when four (W1–W4) or all five water molecules have been targeted. The higher difficulty in partial replacement of the water network by the apolar probes can be understood in light of the higher polarity of the ATAD2 binding pocket compared to other bromodomains.^75^

While the high sequence similarity of the binding pockets of the bromodomains makes it difficult to distinguish the network stability profiles based on the static protein structures, our results suggest that dynamics, including possible long-range rearrangements due to differences in residues located further away from the binding site, play a role in different interaction patterns with the water molecules. These findings emphasize the usefulness of methods that incorporate entropic contributions into the description of the process.

Although the performed perturbations are a significant simplification of the chemical complexity of small-molecule ligands, these observations show qualitative agreement with the known displaceability of these waters. Co-crystal structures of the more widely studied BRD4(1) protein show ligands that partially displace the water network, overlapping with the calculated volume occupancies of W1, W1 and W2, or W1–W3^65,70,76−78^ (Figure S17a). The results exhibited here suggest that there are opportunities for partial network replacement available to BRD3(1) and BRD4(2) as well. In contrast, to the best of our knowledge, ATAD2 does not have any known ligands that displace a single or a reduced number of waters from the network, with the exception of a fragment of low affinity that displaces W4.^79^ Higher affinity ligands either do not interfere with the conserved water molecules or completely displace waters 1–4^63,80^ (Figure S17b), in agreement with the calculated higher free-energy costs for partial network replacement. The favorability of the perturbations when W5 is also replaced suggests a strategy for further ligand optimization for this traditionally difficult to target protein.

An easier displaceability of the full water network in ATAD2 was also suggested in GCI simulations.^67^ This was interpreted as indicative of a highly unstable water network as compared to other bromodomains. The results presented here extend this interpretation. The high unfavorable free energies for individual water replacements indicate that ATAD2 does not necessarily have a weakly bound water network. Rather, the results suggest the existence of more pronounced solvation correlation effects that make the complete replacement of the water network preferable over partial replacements, unlike Family II proteins, which have a higher number of possible perturbations to the network that are energetically accessible. This solvation-dependent stability may also be the reason for the historically low tractability of ATAD2 within the bromodomain class, with even computational druggability assessments based on combinatorial searches of hydration sites in the apo pocket deeming it the most undruggable of the bromodomain proteins.^66^ Such network correlation effects may also be behind surprising complete displacements of the conserved waters by selective inhibitors of other bromodomains, such as CECR2, which were initially predicted to have some of the most stable water networks.^63^

Although other trends from the GCI work^67^ are recapitulated by the RE-EDS simulations as well, such as the lower stabilities of W1 and W4 compared to the other sites in ATAD2 (here indicated by the low ΔΔ*G*_replacement_), some discrepancies are observed. For example, in ref (67), the relative water free-energy scores for water molecules 1–4 (W5 was not considered) point to a lower stability of the waters in BRD4(1) as compared to the other proteins in Family II, especially for W1 and W4. We reason that the differences arise from two factors. First, the two computational approaches consider different processes, since the RE-EDS results refer to the favorability of replacement of the water molecules by an apolar probe, and not the stability of the waters relative to a situation where the pocket is “dry”. Second, GCI calculates rigorous free energies for the binding of waters to the whole GCMC insertion/deletion region. In order to calculate the stability of the conserved network and of the individual water molecules, the resulting free energy was decomposed into estimated scores based on the volume occupied by those waters in the GCMC region. The network free-energy score and the stability of combinations of hydration sites, then, correspond to the sum of the individual contributions of the water molecules, which by definition neglects solvation correlation effects upon joint perturbations to the water network.

### Network Analysis Highlights Differences in Solvation Correlations

**Figure 7:**
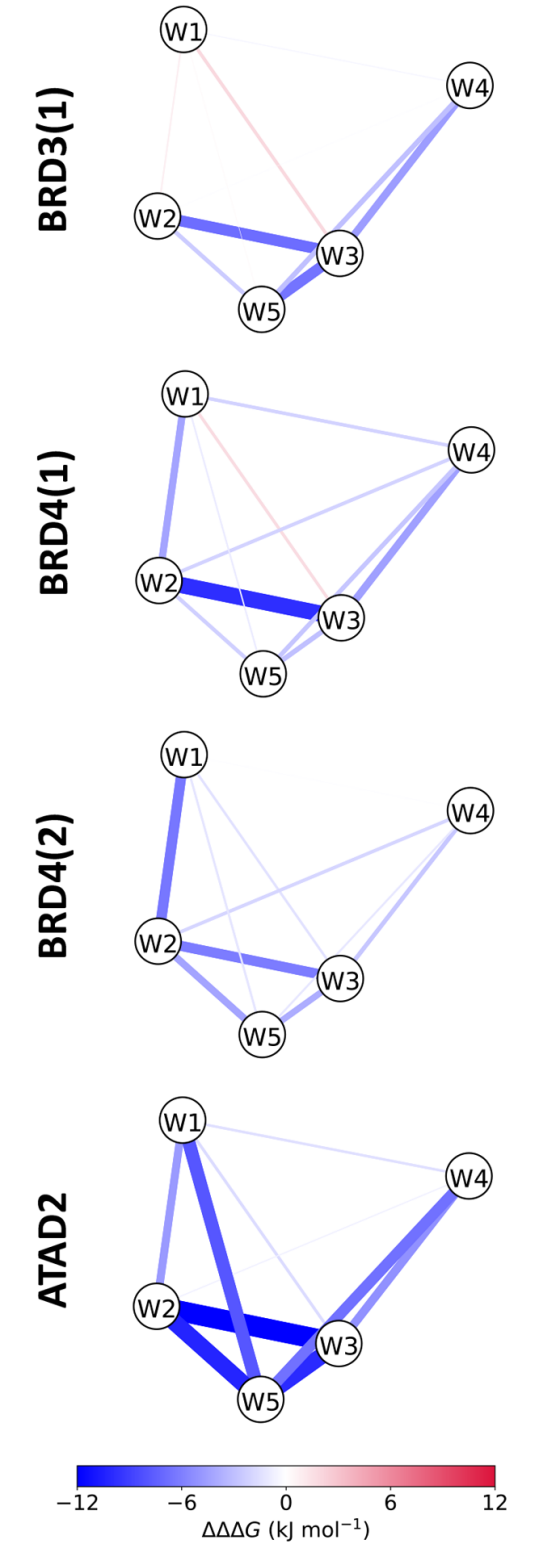
Graph representation of the pairwise correlations between the hydration sites in the bromodomain proteins. Edges are colored according to the change in ΔΔ*G*_replacement_ of one of the water nodes upon replacement of the other water node. Blue lines indicate positive solvation correlation among the water sites (that is, replacement of one of the water molecules results in a more favorable replacement of the other), while red lines indicate negative solvation correlations. Edge thickness is proportional to |ΔΔΔ*G*_replacement_|.

The water replacement free energies obtained at different conditions of the pocket environment allow for a more detailed study of correlation and cooperativity within water networks. We decided to investigate this by looking at the correlation effects of individual water replacements. Analysis of the changes to the ΔΔ*G*_replacement_ upon single perturbations to the water network (Table S6) indicate that, in accordance with the distinct free-energy profiles, the conserved water molecules of the studied bromodomains show different degrees of solvation correlation (Figure 7). Several of the hydration positions are positively correlated (blue lines), indicating that replacement of a water molecule contributes favorably to a subsequent replacement of the nearby water, reducing the free-energy cost. For example, for BRD4(1), replacement of W1 makes the subsequent targeting of the neighboring W2 easier, which can help explain the occurrence of inhibitors that displace this combination of water molecules. In support of the hypothesis of more pronounced solvation correlation effects in ATAD2, stronger positive correlations among all water sites can be observed for this protein.

Interestingly, the existence of weak negative network correlations is suggested for BRD3(1) and BRD4(1). The replacement of W1 further stabilizes W3, and vice versa, leading to an increase in ΔΔ*G*_replacement_. BRD3(1) shows additional, although weaker, negative correlations between W1 and W2, and W1 and W5. This is opposite to what might be expected from a general analysis of the crystallographic structure and considerations about changes to the polarity of the pocket environment, but could be one of the factors behind the challenges of designing BRD3(1) and BRD4(1) inhibitors that further replace the conserved waters.

## Conclusions

Water thermodynamic contributions are at the core of all biological processes. The thermodynamic mapping of water sites has become a tool in rational drug design and lead optimization, with a diversity of computational methods aiming to provide an energetic characterization of water molecules in the protein binding site that cannot be accessed by experimental methods or direct observations of the protein structure. Despite their popularity, these methods predict water energetics at a fixed composition of the environment, usually the apo pocket, without considering the effects of perturbations to the water network as the initial hit compound progresses in the lead optimization pipeline.

Here, we explicitly account for solvation correlation effects through the application of the RE-EDS multistate free-energy method for the combinatorial replacement of hydration sites in the protein binding pocket. Investigations of the BPTI and bromodomain proteins indicate that the likelihood of water replacement is highly dependent on the pocket environment; thus, the water stabilities predicted from an apo pocket do not provide the full picture. Interestingly, we found that for all of the cases studied, the free-energy difference of simultaneous replacement of all waters is always less than the sum of the replacement free energies of the individual water molecules (Figure 4b, Figure 5b, and Table 2), highlighting the penalties associated with partial disruption of water networks in protein binding sites. As organized and evolutionary conserved water networks are widespread in biologically relevant protein targets, we believe that such careful considerations of solvation correlation effects can be beneficial for the rational design of lead compounds. We found that the penalty for replacing the binding site waters varies between members of the bromodomain family and is in qualitative agreement with their displaceability based on known inhibitors, suggesting opportunities for selective inhibitor design through the exploitation of the conserved water network.

**Table 2:** Replacement Free Energies of the Water Network (kJ mol^–1^), Computed via the Sum of the Values of the Individual Water Molecules (ΔΔ*G*_replacement,(i)_) or by Simultaneous Replacement of the Network (ΔΔ*G*_replacement,all_)

System	*∑* ΔΔ*G*_replacement,(i)_	ΔΔ*G*_replacement,all_
BPTI	22.0 ± 2.1	5.5 ± 0.6
BRD3(1)	20.3 ± 3.3	–1.2 ± 1.9
BRD4(1)	21.6 ± 3.5	–6.9 ± 2.0
BRD4(2)	18.9 ± 3.4	–0.4 ± 3.2
ATAD2	45.2 ± 4.0	–3.6 ± 2.8

The alterations to the RE-EDS parameter optimization scheme introduced here enable the efficient application of the method for the thermodynamic mapping of water sites in protein pockets, explicitly considering solvation correlation effects. The methodology does not require restraining of the protein dynamics, such that entropic contributions of the protein to the solvation energetics are also considered. RE-EDS has been implemented in both GROMOS^44,46^ and OpenMM,^48^ and the RE-EDS pipeline is freely available at https://github.com/rinikerlab/reeds.

## Data Availability

The RE-EDS module for parameter optimization is freely available on Github at https://github.com/rinikerlab/reeds. All input protein coordinate and topology files for the simulations can be obtained at https://github.com/rinikerlab/reeds/tree/main/examples/systems/water_replacement, as well as example input files for the RE-EDS simulations.

## Supporting Information Available

Theory of EDS and RE-EDS calculations, adaptations of the RE-EDS parameter estimation workflow for free-energy calculation of water replacement, methodological details for the RE-EDS and RE-TI calculations, and supporting figures and tables referenced in the text (PDF)
