# Supplementary material for: Accounting for Solvation Correlation Effects on the Thermodynamics of Water Networks in Protein Cavities
Source: J Chem Inf Model. 2023 Mar 14;63(6):1794–805. doi: 10.1021/acs.jcim.2c01610 (PMC10052353; doi:10.1021/acs.jcim.2c01610)
Supplement: Supplementary file 1 — ci2c01610_si_001.pdf [file ci2c01610_si_001.pdf]

SUPPORTING INFORMATION
Accounting for Solvation Correlation Effects on the Thermodynamics of Water Networks in Protein Cavities

Emilia P. Barros,[a] Benjamin Ries,[a] Candide Champion,[a] Salomé R. Rieder,[a] and Sereina Riniker[a]*

[a] *Laboratory of Physical Chemistry, ETH Zürich, Vladimir-Prelog-Weg 2, 8093 Zürich, Switzerland.*
*E-mail: sriniker@ethz.ch

1 Theory

1.1 Free-Energy Methods: EDS and RE-EDS

The EDS free-energy method allows for the calculation of free-energy differences between multiple end states from a single simulation by the construction of a reference state Hamiltonian that envelops the Hamiltonians of the end states, without the need of specifying an alchemical path that connects them.[1–4] For N end states, the potential-energy function of the EDS reference state R is defined as:

$$V_R(\vec{r};s,\vec{E}^R) = -\frac{1}{\beta s}\ln\left[\sum_{i=1}^{N} e^{-\beta s\left(V_i(\vec{r})-E_i^R\right)}\right], \tag{1}$$

where $\beta = (k_\mathrm{B}T)^{-1}$, with k_B as the Boltzmann constant and T the absolute temperature.

Two sets of parameters, the smoothing parameter s and the energy offsets $\vec{E}^R$ are tuned to enable optimal sampling of the end states.[1, 2, 4, 5] The energy offsets align the minima of the potential energy surface of the end states such that all contribute equally to the reference potential, while the smoothing parameter decreases the energy barriers between the potential-energy minima corresponding to each end state, such that transitions can occur within the reference state (Figure 1b). In the replica-exchange EDS (RE-EDS) method, the introduction of Hamiltonian replica exchange in the s space reduces the search space to the selection of an appropriate range and distribution of the s replicas, besides the estimation of the energy offsets.[6, 7]

Replicas at low s-values are important to facilitate transitions between each end state's relevant regions of phase space. Regions of small s lead to an unphysical minima in which all end states contribute to the reference state and collectively form what is referred to as the "undersampling regime". [4, 5] In replicas at higher s-values, the end state having the largest impact on the reference state at each timestep in the simulation can be identified by the maximum contribution metric, which is defined, based on Eq. (1), as the end state with the lowest value for the difference between its potential energy in the reference state ensemble and energy offset.[5] Ideal sampling corresponds to the situation in which the fraction of maximum contribution f_i^{mc} is equal for all states being considered, that is,

$$f_i^{\mathrm{mc,ideal}} = \frac{1}{N}, \forall\, i \in \{1,...,N\} \tag{2}$$

The final free-energy differences between each pair of states is then calculated from the physical s=1 replica by applying Zwanzig's equation[8] twice, [1, 2] forming a path between states A and B via the reference state R:

$$\begin{aligned}\Delta G_{\mathrm{BA}} &= \Delta G_{\mathrm{BR}} + \Delta G_{\mathrm{RA}} \\ &= -\frac{1}{\beta}\left(\ln\langle e^{-\beta(V_B-V_R)}\rangle_R - \ln\langle e^{-\beta(V_A-V_R)}\rangle_R\right) \\ &= -\frac{1}{\beta}\ln\frac{\langle e^{-\beta(V_B-V_R)}\rangle_R}{\langle e^{-\beta(V_A-V_R)}\rangle_R} \end{aligned} \tag{3}$$

1.2 Adaptation of the RE-EDS Workflow for Water Replacement Free Energies

Sampling of the relevant regions of phase space of each end state by the EDS reference state is ensured by selection of appropriate s and $\vec{E}^R$ parameters. The automated pipeline developed by Ries *et al.* [5] contains five steps prior to production for the estimation and optimization of the required parameters for RE-EDS simulations. We hypothesized that the large phase-space overlap between the different end states and the reduced complexity of the perturbations would allow for a reasonable simplification of the parameter estimation process. In accordance with this, tests on the protein targets evidenced that a default range of s values can be provided instead of performing a system-specific estimation of the lower s-bound: 21 logarithmically-distributed replicas between 0.00178 and 1 provide a good starting point and are sufficient to guarantee undersampling (Figure S1). The large overlap between states results in the occurrence of replica exchanges even in the non-optimized s-distribution, such that no replica exchange bottlenecks, as is common for ligand perturbations ,[5–7] occur (Figure S2).

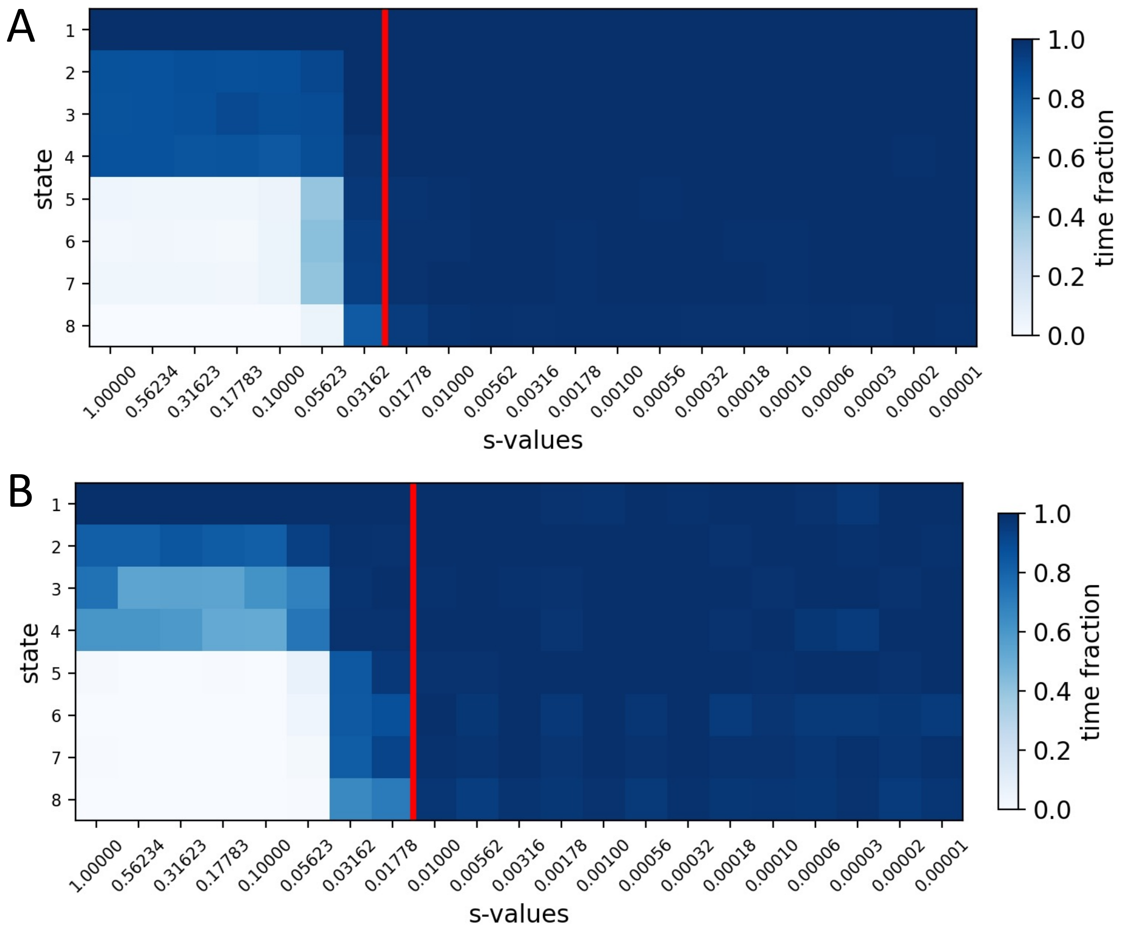

Figure S1: Undersampling and lower *s*-bound (red) for the BPTI simulations in (**A**) bulk water and (**B**) protein environment. The matrix is colored according to the time fraction undersampling occurs according to the criteria in Ries *et al.* [5]

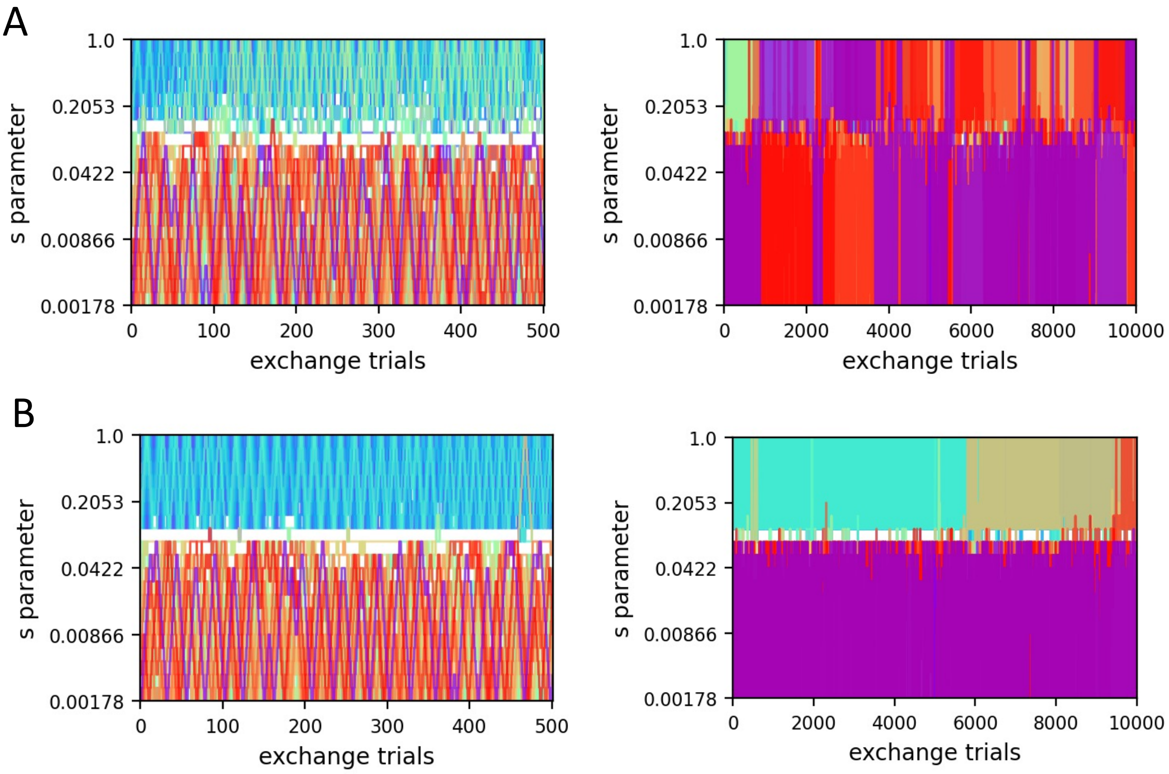

Figure S2: Replica exchanges in (**A**) bulk water and (**B**) BPTI protein RE-EDS simulations with logarithmically distributed replicas. For easier visualization, exchange traces for the first 500 trials are shown in the left, while results for the whole simulation length are shown in the right.

Further simplification was introduced in the energy offsets calculation stage. Based on analysis of the optimized $\vec{E}^R$ for our test systems, we introduced a simpler and more efficient approach for an initial estimate of the parameters according to the number of apolar probes N_{probes} present at each end state:

$$E_i = N_{probes} \cdot 35 \text{ kJ mol}^{-1} \tag{4}$$

Since the pipeline includes a subsequent step for iterative rebalancing of the energy offsets according to sub-optimal state sampling in short RE-EDS simulations, the approximation introduced by this simplistic estimation is not problematic.

With the absence of exchange bottlenecks, the global round-trip optimization algorithm (N-GRTO) is used for the s-optimization step, where the replicas are re-distributed in s-space according to areas of reduced exchanges.[7] As no significant exchange bottlenecks were encountered in the default s-distributed replicas, it would be possible to remove this step altogether from the workflow, but a significant improvement in replica-exchange frequencies was observed for the simulations in the protein environment after a single iteration of s-optimization (Figure S3).

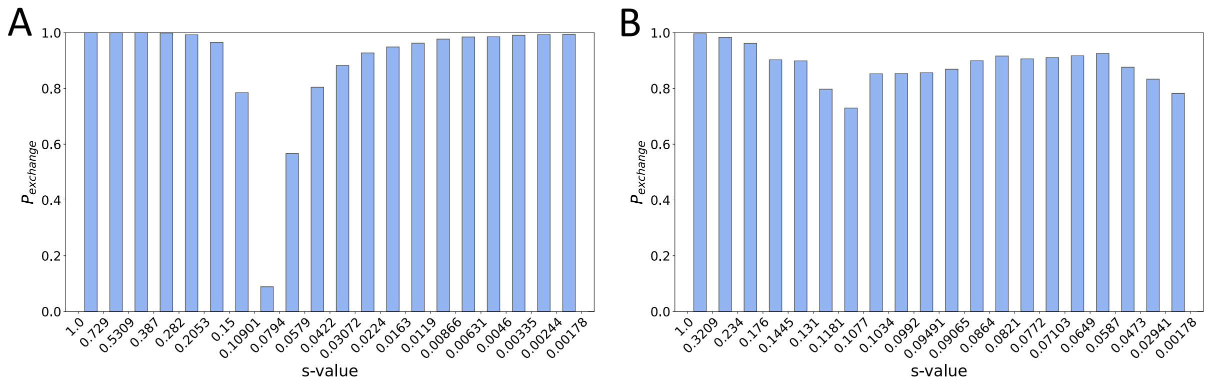

Figure S3: Probabilities of replica exchange in a RE-EDS simulation of the BPTI protein system (a) with default s-distribution and (b) following optimization of the replicas.

While all other steps could be reasonably simplified from the original workflow, our investigations showed that rebalancing of the energy offsets is crucial to ensure sampling of all end states. In this step, the $\vec{E}^R$ are iteratively refined based on the state sampling obtained from short simulations, corrected according to the deviation of each state maximum contribution sampling to the ideal $f_i^{\text{mc,ideal}}$:[5]

$$\Delta E_i^{\text{corr}} = -\frac{1}{\beta} \ln \left(\frac{f_i^{mc} + c}{f_i^{mc,ideal} + c} \right) \tag{5}$$

The pseudo count c is introduced to avoid singularities in the case of zero sampling, defining a minimum x-fold reduced sampling compared to the ideal sampling case:

$$c = \frac{f_i^{mc,ideal}}{x} \tag{6}$$

The degree of corrections to the energy offsets in an iteration can thus be modulated by the choice of the intensity factor x. A high intensity factor might allow for a faster initial correction of poorly chosen energy offsets, but potentially introduces oscillations in further refinement iterations, such that it is not possible to say *a priori* which intensity factor will be most appropriate for a particular system. Instead, we take advantage of the fact that different distributions of state sampling are reached by the use of distinct intensity factors to perform triplicate production simulations with the $\vec{E}^R$ taken from three distinct, parallel runs of energy offset rebalancing using $x = 2$,5, and 10 (Figure S4). The minimum deviation from ideal sampling, as well as the maximum diversity on the identity of the poorly-sampled states between the replicates, are used to determine the iterations that are carried on to production. This leads to a more stringent measure of the variance and confidence of the results than performing replicates with different initial velocities starting from the same simulation conditions, and can reduce inaccuracies in the free-energy estimates due to poor sampling of specific states, if they are better sampled in a different replicate. With these changes, the RE-EDS workflow for water replacement investigation can be simplified as shown in Figure S5.

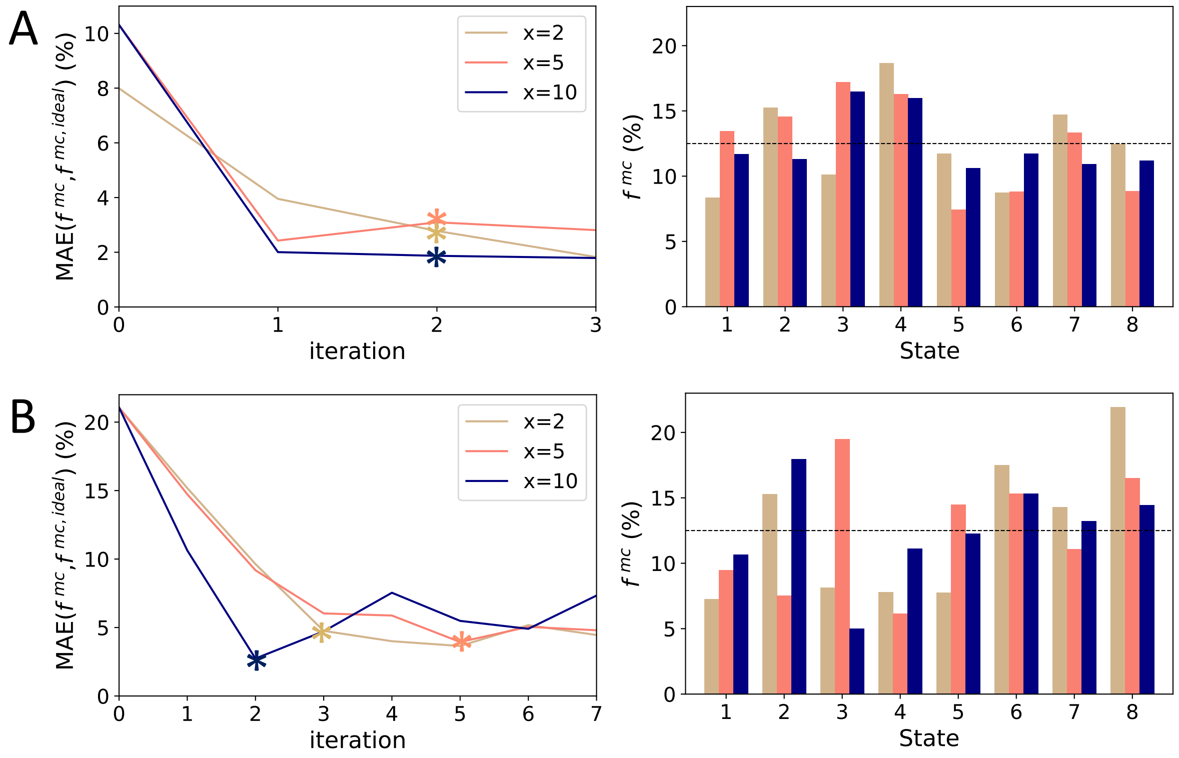

Figure S4: Mean absolute deviation (MAE, in percentage) of the observed state sampling f_i^{mc} from the ideal distribution $f_i^{\mathrm{mc,ideal}}$ at different iterations of energy offset rebalancing (left) and state sampling at the iterations indicated with asterisks using distinct intensity factors (right) for (a) bulk water and (b) protein simulations for the BPTI system. The dotted line indicates $f_i^{\mathrm{mc,ideal}}$.

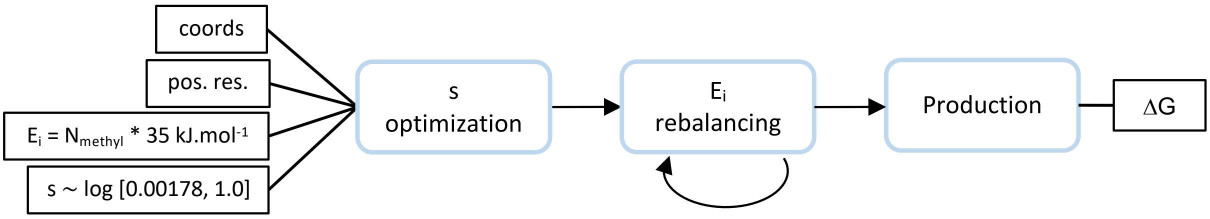

Figure S5: RE-EDS workflow for water replacement free energies. For the simulations in bulk water, the *s*-optimization step can be skipped altogether.

2 Computational Methods

2.1 Simulation Details

Simulations were carried out with the GROMOS software package [9] and the GROMOS 54A7 force field, [10] employing the leapfrog scheme [11] with a time step of 2 fs. Non-bonded interactions were treated with a twin-range cutoff scheme with cutoffs of 0.8 and 1.4 nm, and electrostatic interactions beyond the long-range cutoff were treated with a reaction-field force [12] with a relative dielectric permittivity of 66.7.[13, 14] Bond lengths were constrained with SHAKE [15] with a relative tolerance of 10^{-4}, and the translational motion of the center of mass was removed every 1000 steps. Temperature was controlled at 300 K with the weak-coupling scheme [16] and a relaxation time of 0.1 ps. Pressure was kept close to 1.013 bar (1 atm) with a weak coupling to a pressure bath with a relaxation time of 0.5 ps and an isothermal compressibility of $4.5 \cdot 10^{-4}$ (kJ mol^{-1} nm^{-3})$^{-1}$.

2.2 System Preparation

BPTI coordinates were taken from PDB ID 5PTI,[17] while the protein coordinates in 2NXB (chain A),[18] 5I80,[19] 2OUO [18] and 4QUT [20] were used for BRD3(1), BRD4(1), BRD4(2) and ATAD2, respectively. Missing heavy atoms were added with *tleap* (AmberTools16), [21] and protonation of ionizable residues assigned with Schrödinger Maestro (version 12.5.139, release 2020-3, Schrödinger, LLC, New York, NY) according to a pH of 7.0. All crystallographic waters were kept, and structures were solvated with the simple-point-charge (SPC) water model [22] in rectangular boxes with a minimum wall distance of 0.8 nm. Solvent energy minimization was performed with the steepest descent algorithm, keeping the solute atoms positionally restrained with a force constant of 25'000 kJ mol^{-1} nm^{-2}. Chloride or sodium ions were added to neutralize the system.

Equilibration and thermalization was performed in five consecutive steps in the NVT ensemble, heating from 60 to 300 K for a total of 100 ps, with the force constant for solute restraining sequentially decreased by one order of magnitude at each step. To allow for solvent relaxation and to select the starting structure for RE-EDS, a short unconstrained 5-ns production simulation was performed under NPT conditions. A frame which showed waters with good overlap with the water network in the crystal structure based on visual observation (three waters for BPTI and five waters for the bromodomains, respectively) was taken as the starting structure for the free-energy calculations. The input coordinate files can be obtained at `https://github.com/rinikerlab/reeds/tree/main/examples/systems/water_replacement`.

2.3 RE-EDS Calculations

The alchemical water molecules were treated as solute, together with the protein, in the RE-EDS simulations. The coordinates of the network were extracted from the protein pocket and re-solvated in a water box in order to perform the solution simulations from the thermodynamic cycle, retaining the water's relative orientation in the network. The solvent in this new box was also minimized according to the protocol described above. RE-EDS simulations were performed with GROMOS [9] and the open-source Python3 *reeds* module.[5] Weak position restraints were applied to the oxygen atoms of the perturbed waters to allow for localization of the water molecules. A force constant of 500 kJ mol^{-1} nm^{-2} was used for BPTI, while a higher value of 1'000 kJ mol^{-1} nm^{-2} was applied for the bromodomains to avoid disruption of the water network by side chain insertions of the dynamic loop located above the cavity entrance. The same position restraints were employed in the bulk and protein pocket simulations, with the assumption that their contribution is canceled in the final calculated $\Delta\Delta G_{replacement}$ via the use of the thermodynamic

cycle. In order to avoid drifts of the protein relative to the restrained waters, the Cα of a reduced number of residues located in regions of low flexibility were selected for restraining using the same force constants. Three residues were selected for BPTI and four equivalent residues in the structured α helices were selected for the bromodomains, chosen based on low root-mean square fluctuations (RMSF) measured from the 5 ns conventional MD simulations and their location away from the binding pocket (Table S1). Besides these restraints, the rest of the protein was completely flexible and unrestrained in the simulations. The influence of the protein restraints are not explicitly accounted for in the final free energy values, but it is expected that this is of low consequence since the restrained residues have been purposefully chosen based on the local structure rigidity and distance from the binding site where the water replacements are performed.

Table S1: Residue IDs used for restraining and avoiding protein drift in the RE-EDS simulations. ID is given according to the numbering in the prepared structures used for simulation.

System	Residue IDs
BPTI	Arg20, Try23, Cys51
BRD3(1)	Met9, Ile53, Met69, Phe100
BRD4(1)	Leu25, Ile69, Ile85, Phe116
BRD4(2)	Cys9, Ile55, Gly71, Phe102
ATAD2	Leu15, Val56, Leu72, Ala107

A single topology was used for the perturbations, and the GROMOS 54A7 [10] van der Waals parameters for the CH_3 united atom (with charge zero) was used for the apolar probe. The initial s-distribution was taken as 21 logarithmically distributed replicas between $s = 0.00178$ and $s = 1$, and energy offsets determined according to the number of apolar probes in each state as given in Eq. (4). A single 400-ps RE-EDS simulation was performed for s-optimization of the protein simulations (with a previous 400-ps equilibration), and per-replica energy offset rebalancing iterations used the same 400 ps equilibration and production RE-EDS simulations. Rebalancing was performed in triplicate for each system, with intensity factors x = 2, 5 and 10. For the solution simulations, two iterations of energy offset rebalancing were enough in all cases to guarantee nearly-ideal sampling of the states. For the protein simulations, the deviation from ideal sampling and diversity of poorly-sampled states were used to determine the starting points for the production simulations, and are summarized in Table S2. Production simulations were then performed for 6 ns. In all RE-EDS simulations, replica exchanges were attempted every 20 steps. Example input files for the RE-EDS simulations can be found at `https://github.com/rinikerlab/reeds/tree/main/examples/systems/water_replacement`.

Table S2: Number of energy offset rebalancing iterations performed for each replicate protein simulation.

System	Replicate 1, x=2	Replicate 2, x=5	Replicate 3, x=10
BPTI	3	5	2
BRD3(1)	5	7	3
BRD4(1)	4	4	4
BRD4(2)	7	3	4
ATAD2	6	5	5

2.4 RE-TI Calculations

The BPTI replica-exchange thermodynamic integration (RE-TI) calculations used the same protein and solution starting coordinates from the RE-EDS simulations. 21 λ values were used in each case, their distribution determined based on trials starting from equally-spaced values. The final λ-distributions are given in Table S3. RE-TI was performed with GROMOS, [9] with exchanges attempted every 50 steps. Both protein and water simulations were first equilibrated for 0.5 ns, followed by production runs of 1 ns (solution) or 10 ns (protein) in triplicate.

Table S3: λ values used in the RE-TI simulations of BPTI.

Simulation	λ-distribution
Solution	0.0, 0.02, 0.04, 0.06, 0.08, 0.1, 0.15, 0.2, 0.25, 0.3, 0.35, 0.4, 0.6, 0.67, 0.73, 0.8, 0.85, 0.9, 0.95, 0.97, 1.0
Protein	0.0, 0.02, 0.04, 0.06, 0.07, 0.08, 0.1, 0.15, 0.2, 0.25, 0.3, 0.35, 0.4, 0.47, 0.53, 0.6, 0.67, 0.73, 0.8, 0.9, 1.0

2.5 Analysis

The uncertainty of the reported ΔG values corresponds to the standard deviation of the triplicate simulations. The error estimate of the ΔΔG values was calculated by error propagation of the water and protein triplicate's standard deviations. Statistical significance of the correlations of the bromodomains free energy profiles was based on the associated *p*-value, which indicates the probability of obtaining a statistic (in our case, Spearman correlation coefficient) at least as high as the results actually observed, under the assumption of the null hypothesis (i.e., uncorrelated datasets). Considering the relatively small sample size (496 samples), *p*-value was estimated with a permutation test, using the scipy.stats.permutation_test function, with permutation_type set to "pairings" and other parameters taken as default.

RMSD and sequence identity of the bromodomain X-ray crystallographic structures were determined with the MultiSeq [23] plugin in VMD.[24]. Pocket volumes were calculated using POVME, version 3.0. [25, 26] The bromodomain trajectories were aligned to the starting BRD4(1) structure, and the same set of inclusion and seed spheres encompassing the binding site were applied to allow comparison across the bromodomains.

3 Additional Figures Referenced in Main Text

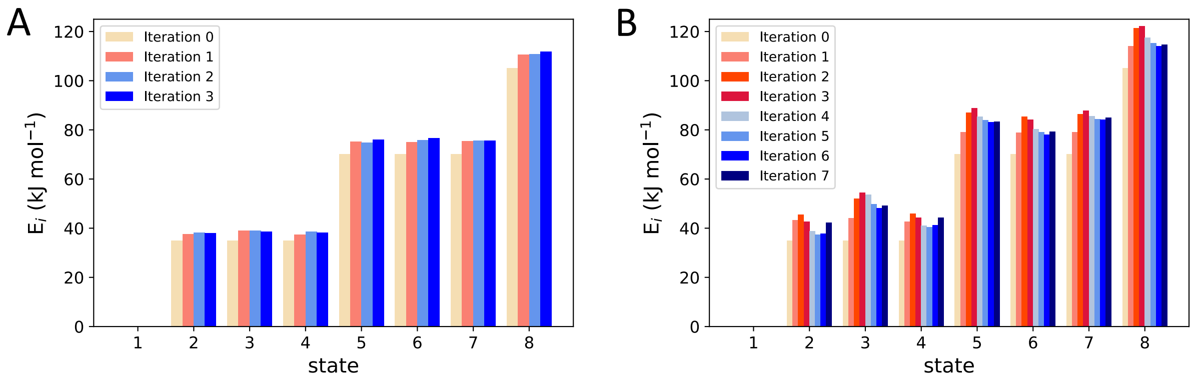

Figure S6: Energy offsets at different rebalancing iterations in the 8-state simulation of BPTI in (**A**) bulk water and (**B**) protein environment. Iteration 0 refers to the results obtained from the initial estimated energy offsets, prior to rebalancing.

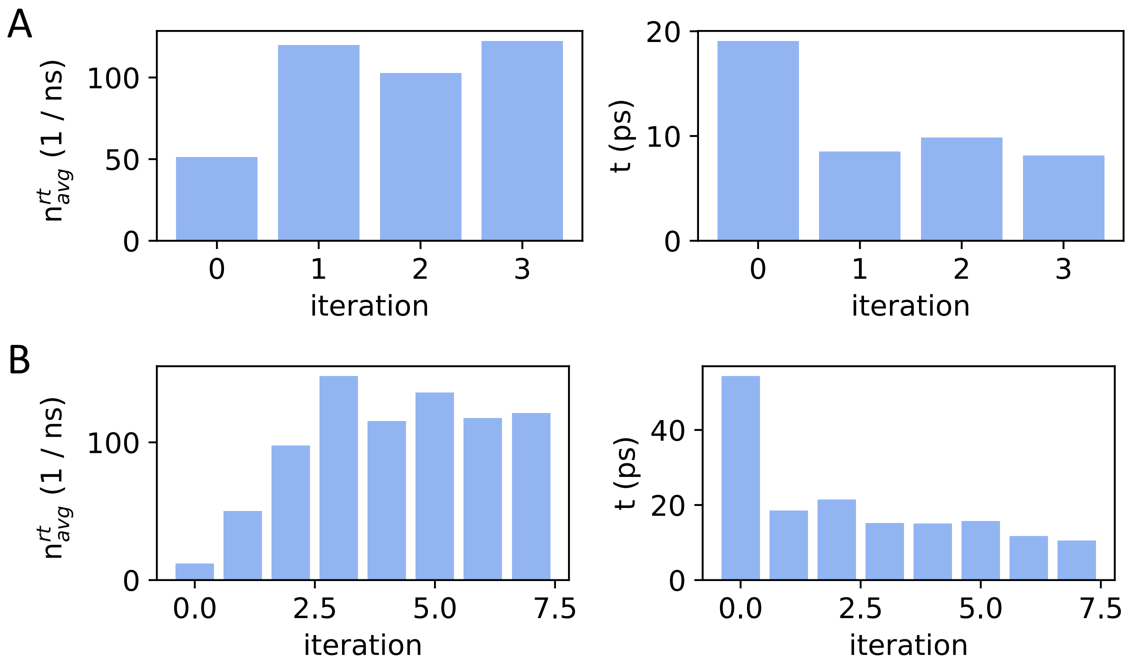

Figure S7: Average number of round-trips (left) and average round-trip time between replicas (right) for the different iterations of energy offset rebalancing for (**A**) bulk water and (**B**) protein BPTI simulations with intensity factor $x = 5$.

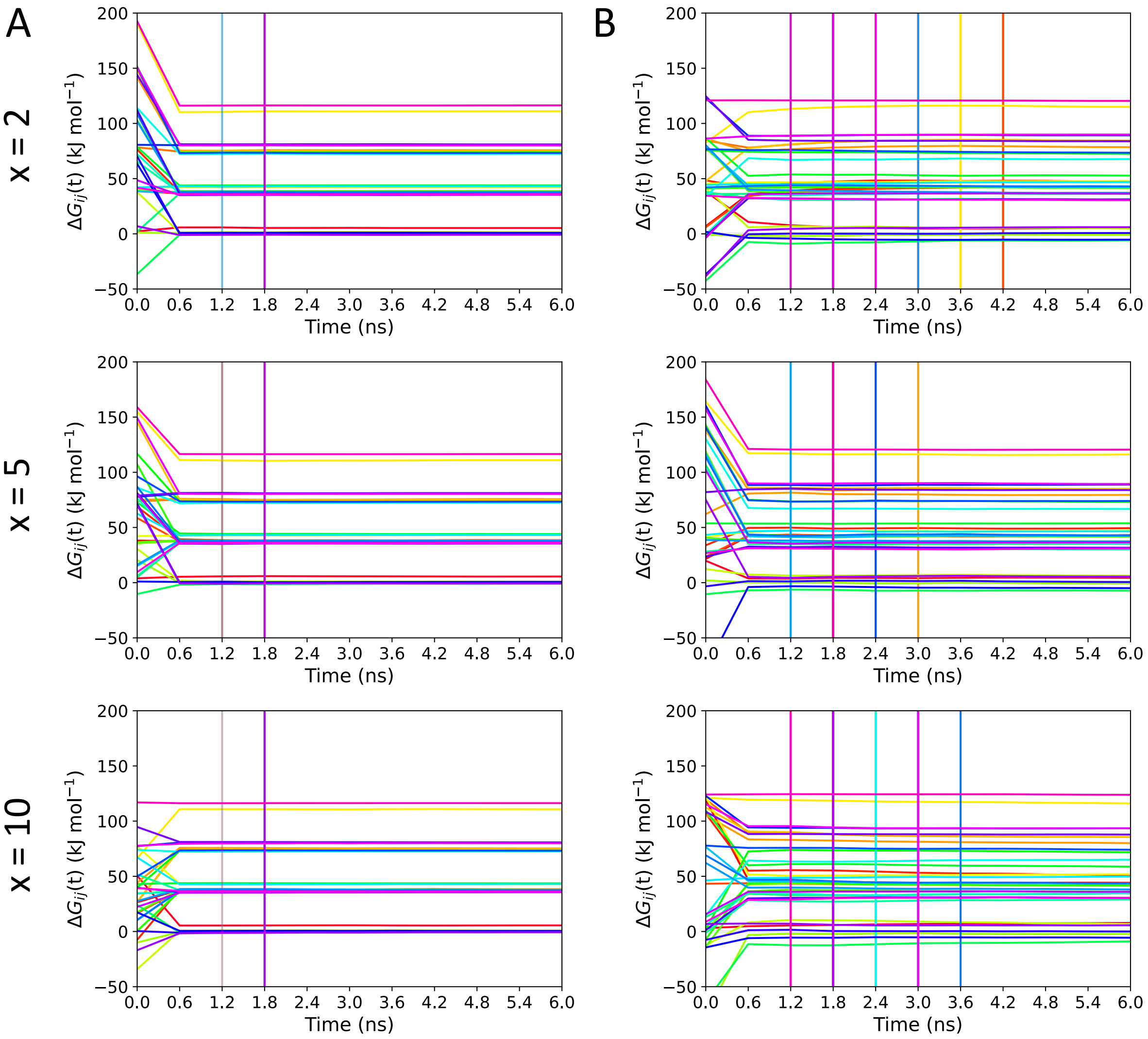

Figure S8: Convergence analysis of the triplicate BPTI simulations in (**A**) bulk water and (**B**) protein environments. The simulations were divided equally into 10 blocks and free-energy values were considered converged when the deviation between three consecutive data points were within 1 kJ mol^{-1} (horizontal lines).

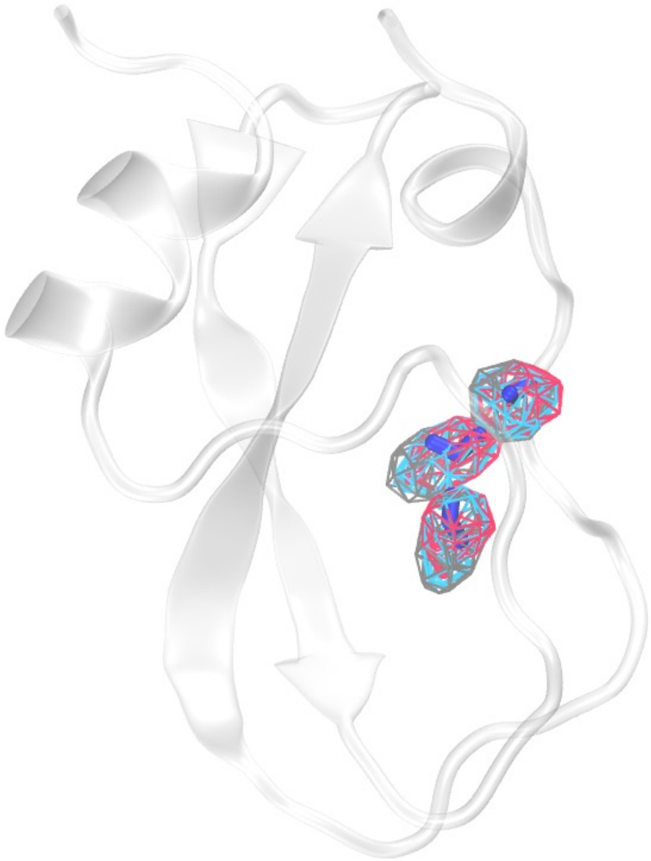

Figure S9: Volume occupancies of the selected water molecules in the triplicate BPTI simulations, according to a cut-off of 20% of the maximum occupancy. Replicate results are shown in grey, pink, and light blue. Water molecules in the starting conformation are represented in blue licorice format.

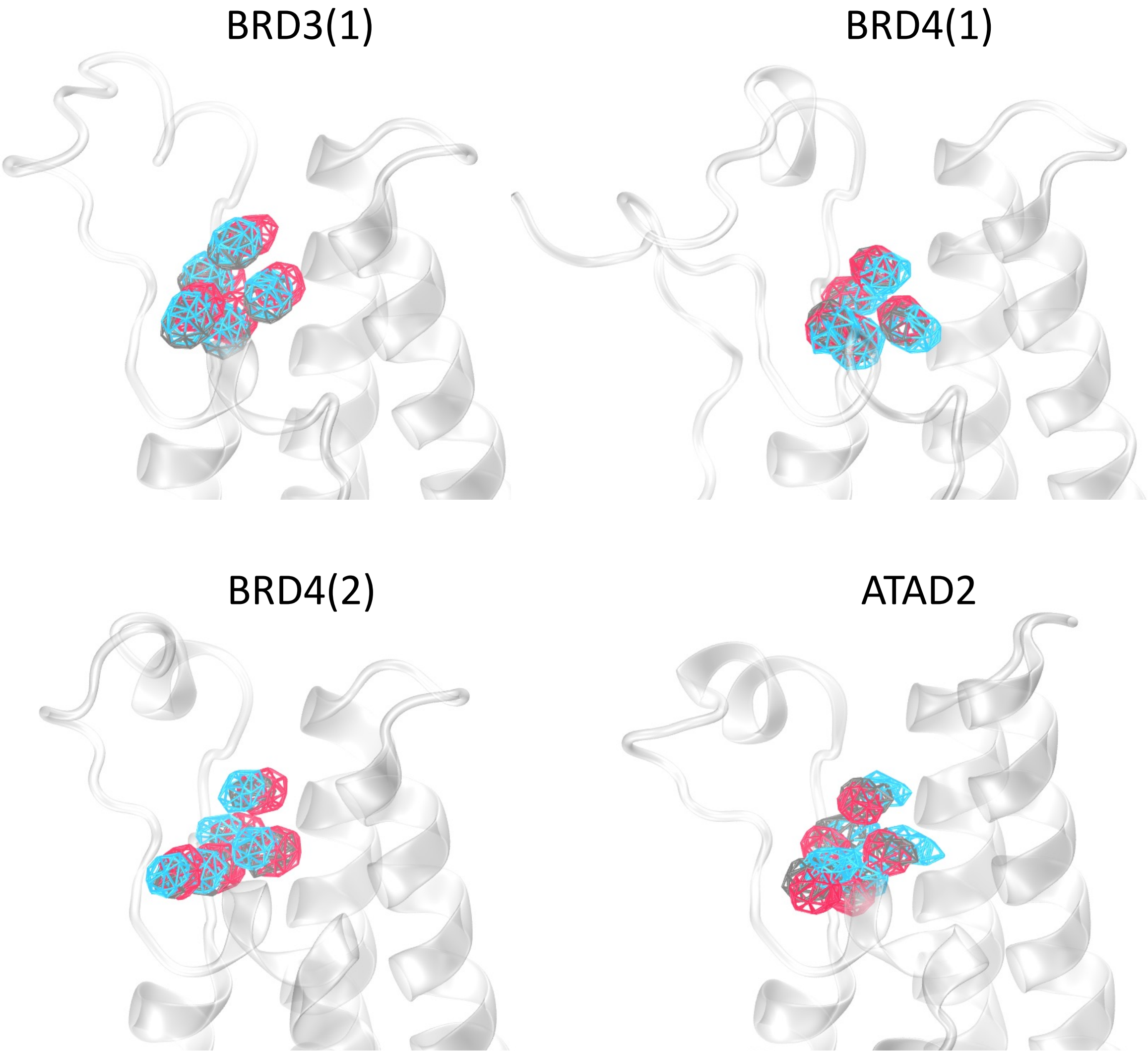

Figure S10: Volume occupancies of the selected water molecules in the triplicate simulations of the bromodomain proteins, according to a cut-off of 20% of the maximum occupancy. Replicate results are shown in grey, pink, and light blue.

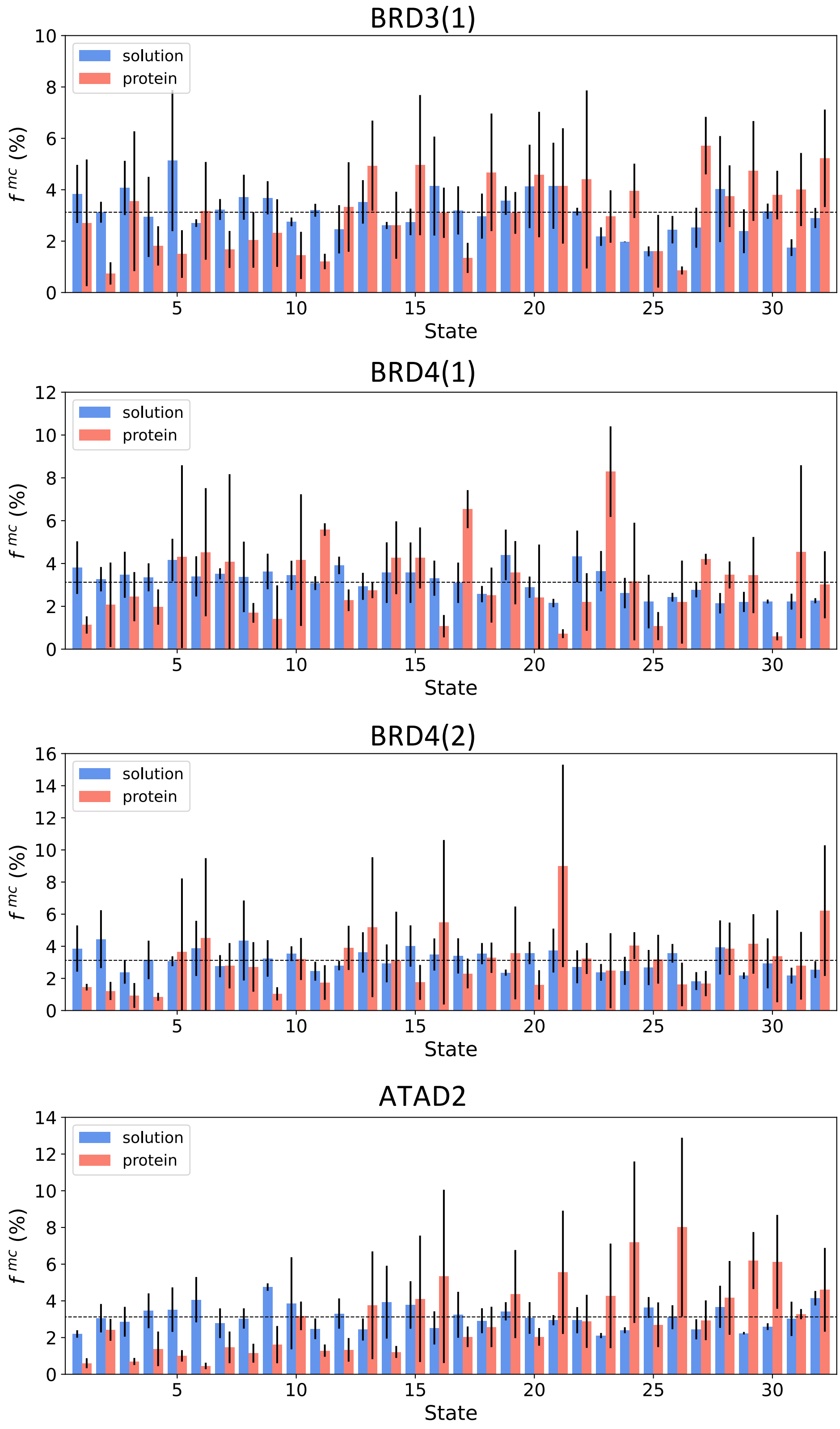

Figure S11: Average state sampling in the triplicate production simulations in bulk water and in the protein environment for the bromodomain systems. Error bars correspond to the standard deviation of the triplicate runs. The dotted line indicates ideal sampling.

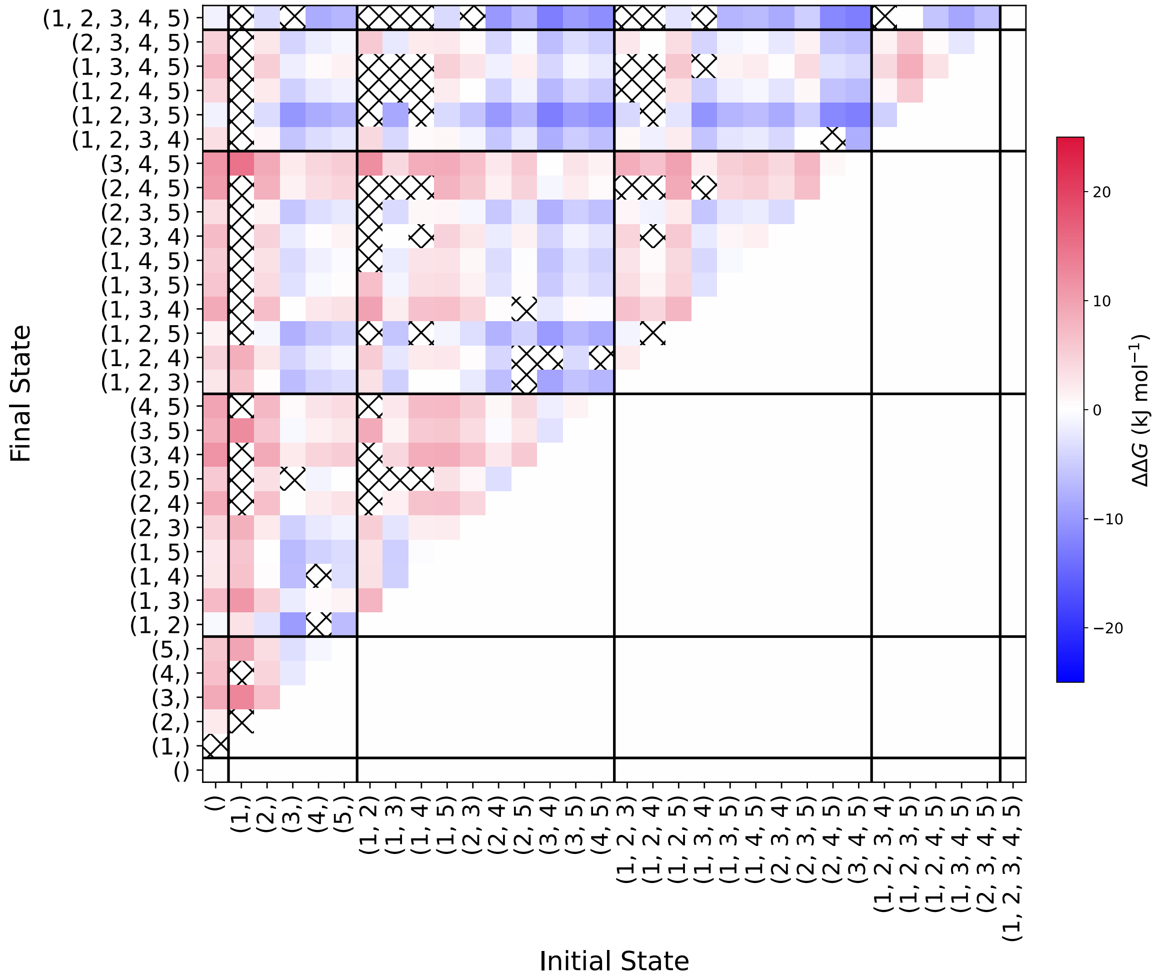

Figure S12: Average ΔΔG results for the BRD3(1) system. Crossed squares in the matrix indicate values for which the standard deviation among the replicates was above 2.5 kJ mol^{-1}. States are labeled according to the hydration site ID occupied by a probe.

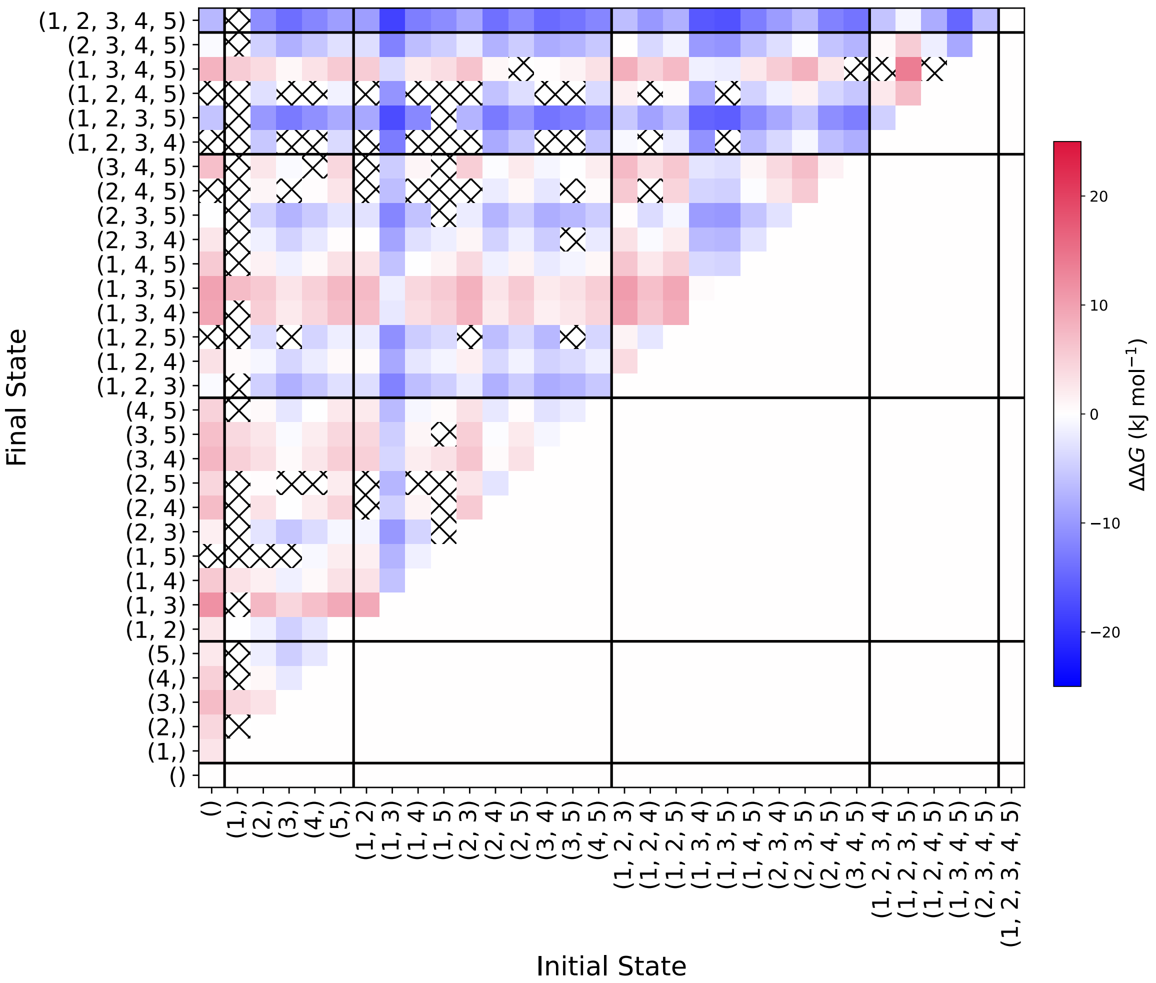

Figure S13: Average ΔΔG results for the BRD4(1) system. Crossed squares in the matrix indicate values for which the standard deviation among the replicates was above 2.5 kJ mol^{-1}. States are labeled according to the hydration site ID occupied by a probe.

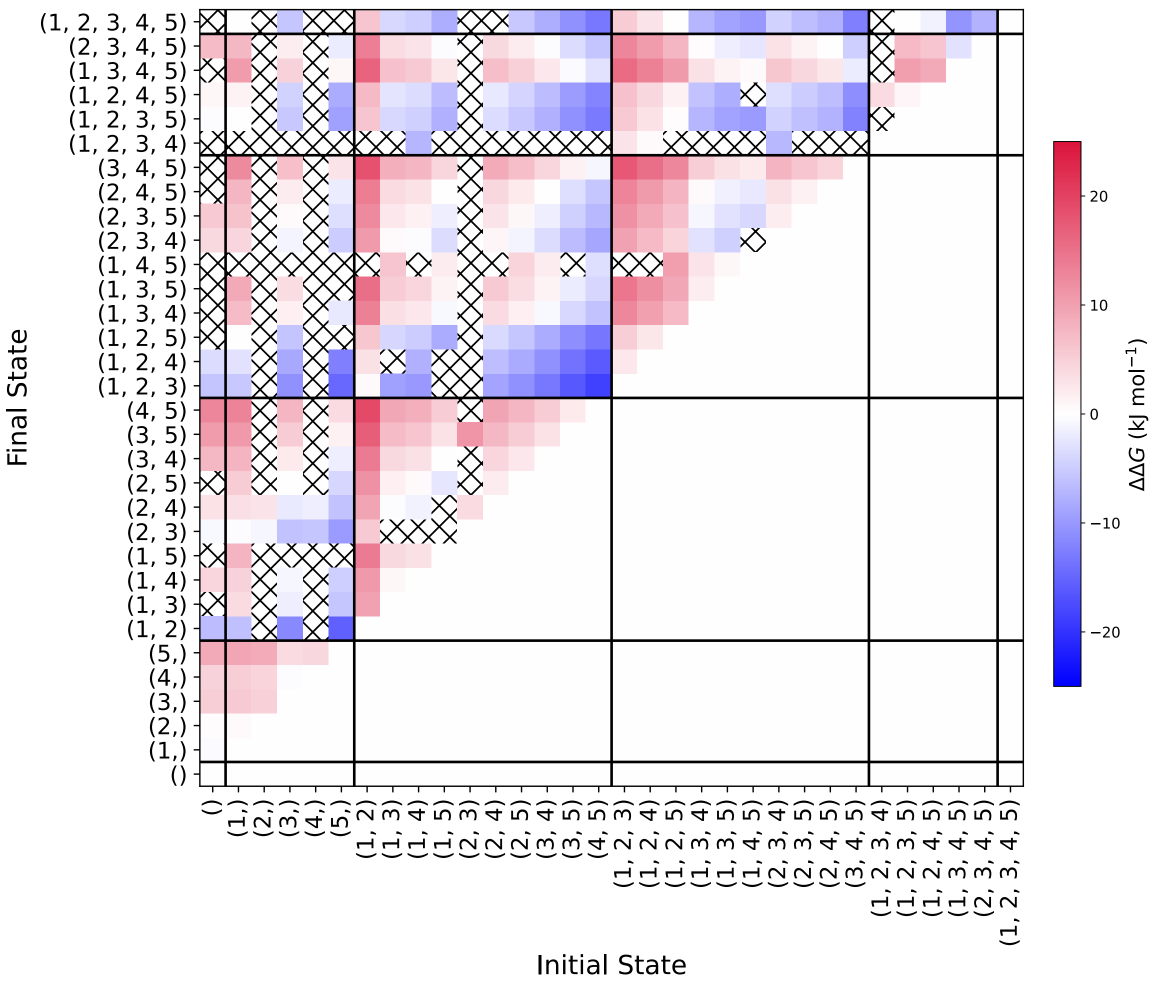

Figure S14: Average ΔΔG results for the BRD4(2) system. Crossed squares in the matrix indicate values for which the standard deviation among the replicates was above 2.5 kJ mol^{-1}. States are labeled according to the hydration site ID occupied by a probe.

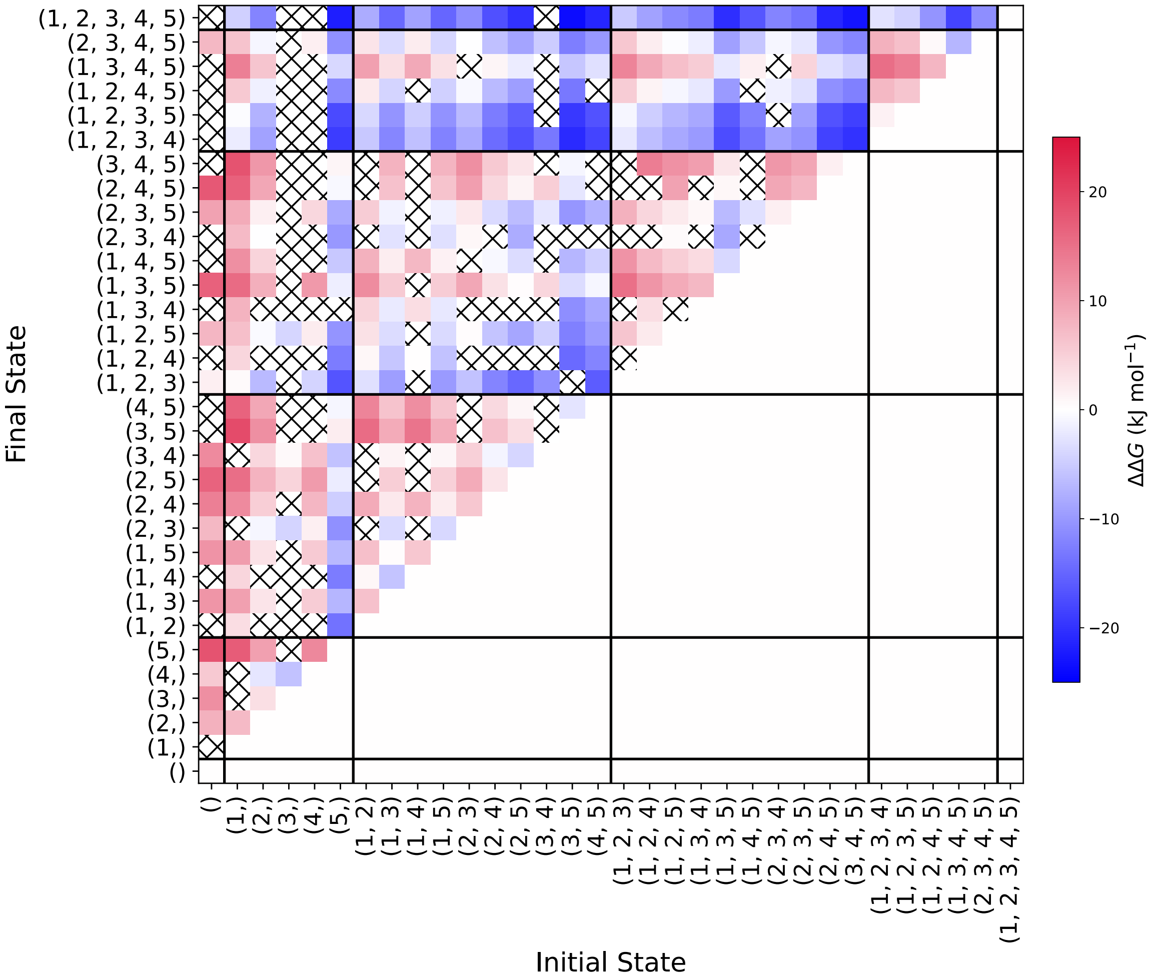

Figure S15: Average ΔΔG results for the ATAD2 system. Crossed squares in the matrix indicate values for which the standard deviation among the replicates was above 2.5 kJ mol^{-1}. States are labeled according to the hydration site ID occupied by a probe.

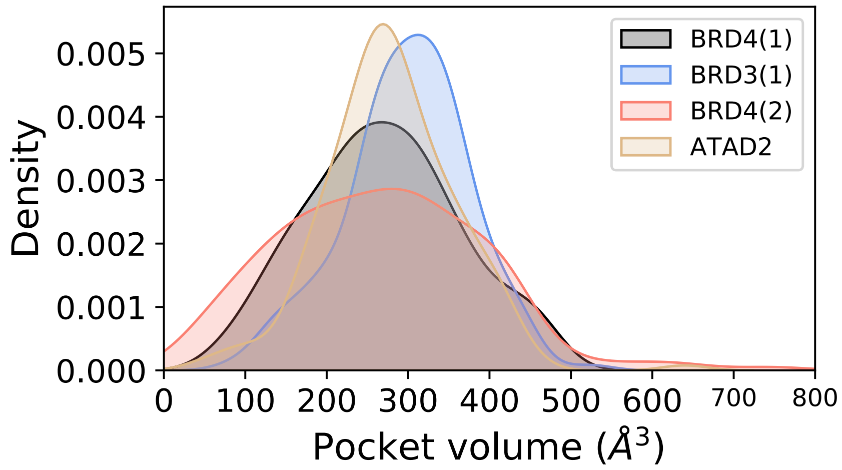

Figure S16: Distribution of pocket volumes from the bromodomain simulations.

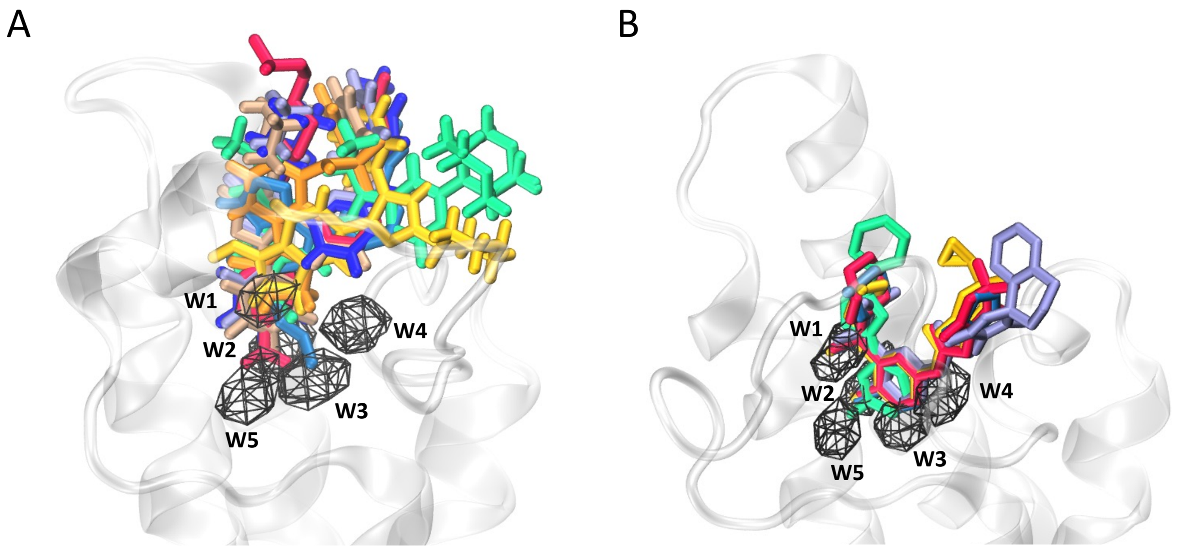

Figure S17: Bromodomain ligands that disrupt the water network of (**A**) BRD4(1) (PDBs 4O7B, 4O7C, 4O7F, 5I88, 5TI2, 6MH7, 6WGX and 6XUZ) and (**B**) ATAD2 (PDBs 4TZ2, 6S55, 6S56, 6S57 and 6YB4) overlayed with the volume occupancies of the selected water molecules studied with RE-EDS.

4 Additional Tables Referenced in Main Text

Table S4: BPTI $\Delta\Delta G_{replacement}$ values (kJ mol^{-1}) from the RE-EDS simulations. Errors indicate standard deviations, calculated by error propagation of the standard deviations of the water and protein triplicate simulations. States are labeled according to the hydration site ID occupied by a probe.

		Final state							
		( )	(1)	(2)	(3)	(1,2)	(1,3)	(2,3)	(1,2,3)
Initial State	( )	-	5.8 ± 1.0	11.4 ± 1.9	4.7 ± 0.2	10.2 ± 0.9	4.3 ± 0.7	10.9 ± 0.6	5.5 ± 0.6
	(1)		-	5.6 ± 1.2	-1.1 ± 0.9	4.3 ± 0.2	-1.5 ± 0.5	5.1 ± 0.7	-0.3 ± 0.5
	(2)			-	-6.7 ± 1.8	-1.3 ± 1.2	-7.1 ± 1.3	-0.5 ± 1.4	-5.9 ± 1.3
	(3)				-	5.4 ± 0.8	-0.4 ± 0.6	6.2 ± 0.5	0.8 ± 0.5
	(1,2)					-	-5.8 ± 0.4	0.7 ± 0.5	-4.6 ± 0.3
	(1,3)						-	6.6 ± 0.3	1.2 ± 0.1
	(2,3)							-	-5.4 ± 0.3
	(1,2,3)								-

Table S5: Relative free energies of replacing all combinations of water molecules from the apo cavity (denoted as state ( )) for the bromodomain simulations. Errors indicate standard deviations, calculated by error propagation of the standard deviations of the water and protein triplicate simulations. States are labeled according to the hydration site ID occupied by a probe.

		$\Delta\Delta G_{replacement}$ (kJ mol^{-1})			
Initial state	Final state	BRD3(1)	BRD4(1)	BRD4(2)	ATAD2
( )	(1)	-3.7 ± 2.6	2.7 ± 1.9	-0.4 ± 1.8	1.0 ± 3.1
	(2)	2.2 ± 1.0	4.2 ± 1.3	0.2 ± 2.2	8.3 ± 1.4
	(3)	9.1 ± 1.2	7.2 ± 0.5	5.2 ± 1.0	11.7 ± 1.1
	(4)	6.8 ± 0.8	5.0 ± 2.2	4.8 ± 1.6	5.8 ± 0.4
	(5)	5.9 ± 0.7	2.5 ± 1.3	9.1 ± 0.5	18.3 ± 1.8
	(1,2)	-0.7 ± 2.2	2.6 ± 1.8	-6.5 ± 1.5	4.6 ± 3.4
	(1,3)	7.2 ± 1.4	11.6 ± 2.0	3.5 ± 2.6	11.1 ± 2.5
	(1,4)	2.6 ± 1.9	5.8 ± 1.6	4.3 ± 1.4	5.4 ± 4.1
	(1,5)	2.4 ± 0.5	4.4 ± 2.9	7.5 ± 3.5	11.4 ± 2.2
	(2,3)	4.5 ± 0.7	1.6 ± 0.8	-0.7 ± 1.9	7.5 ± 0.8
	(2,4)	8.9 ± 0.5	7.1 ± 1.8	3.1 ± 1.0	13.5 ± 1.7
	(2,5)	5.7 ± 1.7	4.5 ± 2.2	5.1 ± 2.6	16.4 ± 1.3
	(3,4)	11.3 ± 0.9	7.7 ± 1.5	7.5 ± 2.3	12.4 ± 0.4
	(3,5)	8.4 ± 0.5	6.7 ± 0.5	10.5 ± 1.5	20.1 ± 3.7
	(4,5)	9.7 ± 0.9	4.8 ± 2.3	12.8 ± 2.4	17.5 ± 3.1
	(1,2,3)	2.5 ± 1.4	-0.5 ± 1.9	-5.7 ± 1.2	1.6 ± 1.8
	(1,2,4)	4.8 ± 2.1	3.2 ± 1.1	-3.3 ± 1.7	5.5 ± 4.0
	(1,2,5)	1.4 ± 1.0	0.8 ± 2.6	-0.6 ± 3.1	7.8 ± 1.7
	(1,3,4)	9.2 ± 1.5	9.4 ± 1.6	6.8 ± 2.9	9.1 ± 4.2
	(1,3,5)	6.1 ± 0.3	9.9 ± 1.2	8.7 ± 3.0	16.7 ± 2.3
	(1,4,5)	5.4 ± 0.5	5.6 ± 2.4	9.6 ± 4.2	12.9 ± 3.1
	(2,3,4)	7.1 ± 0.7	2.7 ± 2.3	4.1 ± 2.3	8.3 ± 2.6
	(2,3,5)	3.6 ± 0.5	-0.2 ± 1.6	5.9 ± 2.0	10.0 ± 2.2
	(2,4,5)	10.5 ± 1.5	5.3 ± 3.3	7.3 ± 3.0	17.7 ± 2.3
	(3,4,5)	11.3 ± 0.3	6.7 ± 1.9	11.9 ± 2.9	19.3 ± 2.6
	(1,2,3,4)	3.4 ± 1.1	-1.1 ± 3.3	-2.9 ± 2.8	-0.7 ± 2.5
	(1,2,3,5)	-1.2 ± 0.8	-5.8 ± 1.4	-0.3 ± 2.3	0.7 ± 2.8
	(1,2,4,5)	4.5 ± 1.3	1.2 ± 3.4	0.9 ± 2.4	6.9 ± 2.7
	(1,3,4,5)	7.4 ± 1.5	8.0 ± 2.0	10.0 ± 2.7	14.6 ± 3.2
	(2,3,4,5)	5.0 ± 0.5	-0.5 ± 1.7	7.1 ± 2.5	7.5 ± 2.3
	(1,2,3,4,5)	-1.2 ± 1.9	-6.9 ± 2.0	-0.4 ± 3.2	-3.6 ± 2.8

Table S6: Relative free energies of replacing the individual water molecules (in kJ mol^{-1}) at different compositions of the water network for the bromodomain systems. Errors indicate standard deviations, calculated by error propagation of the standard deviations of the water and protein triplicate simulations. States are labeled according to the hydration site ID occupied by a probe.

Initial condition	Replaced water molecule W1	W2	W3	W4	W5
BRD3(1)					
( )	-3.7 ± 2.6	2.2 ± 1.0	9.1 ± 1.2	6.8 ± 0.8	5.9 ± 0.7
(1)	-	3.1 ± 1.6	11.0 ± 1.5	6.4 ± 0.8	6.2 ± 2.5
(2)	-2.9 ± 2.0	-	2.3 ± 0.9	6.6 ± 1.4	3.5 ± 2.1
(3)	-1.8 ± 1.1	-4.5 ± 0.9	-	2.3 ± 1.6	-0.6 ± 0.9
(4)	-4.2 ± 2.5	2.1 ± 1.2	4.6 ± 1.4	-	2.9 ± 1.6
(5)	-3.5 ± 0.5	-0.2 ± 2.3	2.5 ± 0.6	3.8 ± 1.2	-
BRD4(1)					
( )	2.7 ± 1.9	4.2 ± 1.3	7.2 ± 0.5	5.0 ± 2.2	2.5 ± 1.3
(1)	-	-0.1 ± 1.7	8.9 ± 2.9	3.0 ± 1.9	1.6 ± 2.9
(2)	-1.5 ± 2.4	-	-2.6 ± 0.9	2.9 ± 0.6	0.3 ± 0.9
(3)	4.4 ± 2.0	-5.6 ± 1.1	-	0.5 ± 1.5	-0.4 ± 0.4
(4)	0.8 ± 0.9	2.1 ± 2.4	2.7 ± 0.9	-	-0.2 ± 1.1
(5)	1.9 ± 2.1	2.0 ± 1.6	4.3 ± 1.6	2.3 ± 1.1	-
BRD4(2)					
( )	-0.4 ± 1.8	0.2 ± 2.2	5.2 ± 1.0	4.8 ± 1.6	9.1 ± 0.5
(1)	-	-6.1 ± 0.6	3.9 ± 1.1	4.7 ± 1.3	7.9 ± 2.1
(2)	-6.7 ± 2.6	-	-0.9 ± 0.5	2.9 ± 2.1	4.9 ± 3.4
(3)	-1.7 ± 1.7	-5.9 ± 1.9	-	2.3 ± 1.4	5.3 ± 0.6
(4)	-0.5 ± 2.9	-1.7 ± 2.1	2.7 ± 3.6	-	8.0 ± 3.7
(5)	-1.6 ± 3.0	-4.0 ± 2.1	1.4 ± 1.0	3.7 ± 1.9	-
ATAD2					
( )	1.0 ± 3.1	8.3 ± 1.4	11.7 ± 1.1	5.8 ± 0.4	18.3 ± 1.8
(1)	-	3.5 ± 1.4	10.1 ± 0.9	4.4 ± 1.6	10.4 ± 1.0
(2)	-3.7 ± 2.6	-	-0.8 ± 0.7	5.2 ± 1.3	8.1 ± 0.4
(3)	-0.6 ± 3.4	-4.2 ± 1.4	-	0.7 ± 1.2	8.4 ± 4.4
(4)	-0.4 ± 4.1	7.7 ± 1.7	6.6 ± 0.3	-	11.7 ± 3.0
(5)	-6.9 ± 1.1	-1.9 ± 1.1	1.8 ± 2.1	-0.8 ± 1.4	-

References

[1] Christ, C. D.; van Gunsteren, W. F. Enveloping Distribution Sampling: A Method to Calculate Free Energy Differences from a Single Simulation. *J. Chem. Phys.* **2007**, *126*, 184110.

[2] Christ, C. D.; van Gunsteren, W. F. Multiple Free Energies from a Single Simulation: Extending Enveloping Distribution Sampling to Nonoverlapping Phase-Space Distributions. *J. Chem. Phys.* **2008**, *128*, 174112.

[3] Christ, C. D.; van Gunsteren, W. F. Simple, Efficient, and Reliable Computation of Multiple Free Energy Differences from a Single Simulation: A Reference Hamiltonian Parameter Update Scheme for Enveloping Distribution Sampling (EDS). *J. Chem. Theory Comput.* **2009**, *5*, 276–286.

[4] Riniker, S.; Christ, C. D.; Hansen, N.; Mark, A. E.; Nair, P. C.; van Gunsteren, W. F. Comparison of Enveloping Distribution Sampling and Thermodynamic Integration to Calculate Binding Free Energies of Phenylethanolamine N-methyltransferase Inhibitors. *J. Chem. Phys.* **2011**, *135*, 024105.

[5] Ries, B.; Normak, K.; Weiss, R. G.; Rieder, S.; Barros, E. P.; Champion, C.; König, G.; Riniker, S. Relative Free-Energy Calculations for Scaffold Hopping-Type Transformations with an Automated RE-EDS Sampling Procedure. *J. Comput.-Aided Mol. Des.* **2022**, *36*, 117.

[6] Sidler, D.; Schwaninger, A.; Riniker, S. Replica Exchange Enveloping Distribution Sampling (RE-EDS): A Robust Method to Estimate Multiple Free-Energy Differences from a Single Simulation. *J. Chem. Phys.* **2016**, *145*, 154114.

[7] Sidler, D.; Cristòfol-Clough, M.; Riniker, S. Efficient Round-Trip Time Optimization for Replica-Exchange Enveloping Distribution Sampling (RE-EDS). *J. Chem. Theory Comput.* **2017**, *13*, 3020–3030.

[8] Zwanzig, R. W. High-Temperature Equation of State by a Perturbation Method. I. Nonpolar Gases. *J. Chem. Phys.* **1954**, *22*, 1420–1426.

[9] Schmid, N.; Christ, C. D.; Christen, M.; Eichenberger, A. P.; van Gunsteren, W. F. Architecture, Implementation and Parallelisation of the GROMOS Software for Biomolecular Simulation. *Comp. Phys. Comm.* **2012**, *183*, 890–903.

[10] Schmid, N.; Eichenberger, A. P.; Choutko, A.; Riniker, S.; Winger, M.; Mark, A. E.; van Gunsteren, W. F. Definition and Testing of the GROMOS Force-Field Versions: 54A7 and 54B7. *Eur. Biophys. J.* **2011**, *40*, 843–856.

[11] van Gunsteren, W. F.; Berendsen, H. J. C. A Leap-Frog Algorithm for Stochastic Dynamics. *Mol. Sim.* **1988**, *1*, 173–185.

[12] Tironi, I. G.; Sperb, R.; Smith, P. E.; van Gunsteren, W. F. A Generalized Reaction Field Method for Molecular Dynamics Simulations. *J. Chem. Phys.* **1995**, *102*, 5451.

[13] Glättli, A.; Daura, X.; van Gunsteren, W. F. Derivation of an Improved Simple Point Charge Model for Liquid Water: SPC/A and SPC/L. *J. Chem. Phys.* **2002**, *116*, 9811–9828.

[14] Riniker, S.; Kunz, A.-P. E.; van Gunsteren, W. F. On the Calculation of the Dielectric Permittivity and Relaxation of Molecular Models in the Liquid Phase. *J. Chem. Theory Comput.* **2011**, *7*, 1469–1475.

[15] Ryckaert, J.-P.; Ciccotti, G.; Berendsen, H. J. C. Numerical Integration of the Cartesian Equations of Motion of a System with Constraints: Molecular Dynamics of n-Alkanes. *J. Comput. Phys.* **1977**, *23*, 327–341.

[16] Berendsen, H. J. C.; Postma, J. P. M.; van Gunsteren, W. F.; DiNola, A.; Haak, J. R. Molecular Dynamics with Coupling to an External Bath. *J. Chem. Phys.* **1984**, *81*, 3684–3690.

[17] Wlodawer, A.; Walter, J.; Huber, R.; Sjolin, L. Structure of Bovine Pancreatic Trypsin Inhibitor. Results of Joint Neutron and X-ray Refinement of Crystal Form II. *J. Mol. Biol.* **1984**, *180*, 301–329.

[18] Filippakopoulos, P.; Picaud, S.; Mangos, M.; Keates, T.; Lambert, J.-P.; Barsyte-Lovejoy, D.; Felletar, I.; Volkmer, R.; Müller, S.; Pawson, T.; Gingras, A.-C.; Arrowsmith, C. H.; Knapp, S. Histone Recognition and Large-Scale Structural Analysis of the Human Bromodomain Family. *Cell* **2012**, *149*, 214–231.

[19] Crawford, T. D. et al. Diving Into the Water: Inducible Binding Conformations for BRD4, TAF1(2), BRD9, and CECR2 Bromodomains. *J. Med. Chem.* **2016**, *59*, 5391–5402.

[20] Morozumi, Y. et al. ATAD2 is a Generalist Facilitator of Chromatin Dynamics in Embryonic Stem Cells. *J. Mol. Cell Biol.* **2016**, *8*, 349–362.

[21] Case, D. A. et al. AMBER Reference Manual. 2016.

[22] Berendsen, H. J. C.; Postma, J. P. M.; van Gunsteren, W. F.; Hermans, J. J. In *Intermolecular Forces*; Pullman, B., Ed.; Reidel: Dordrecht, The Netherlands, 1981; pp 331–342.

[23] Roberts, E.; Eargle, J.; Wright, D.; Luthey-Schulten, Z. MultiSeq: Unifying Sequence and Structure Data for Evolutionary Analysis. *BMC Bioinformatics* **2006**, *7*, 382.

[24] Humphrey, W.; Dalke, A.; Schulten, K. VMD – Visual Molecular Dynamics. *J. Mol. Graph.* **1996**, *14*, 33–38.

[25] Durrant, J. D.; de Oliveira, C. A. F.; McCammon, J. A. POVME: An Algorithm for Measuring Binding-Pocket Volumes. *J. Mol. Graph. Model.* **2011**, *29*, 773–776.

[26] Wagner, J. R.; Sørensen, J.; Hensley, N.; Wong, C.; Zhu, C.; Perison, T.; Amaro, R. E. POVME 3.0: Software for Mapping Binding Pocket Flexibility. *J. Chem. Theory Comput.* **2017**, *13*, 4584–4592.
